# Annexin A2 plays a key role in protecting against cisplatin-induced AKI through β-catenin/TFEB pathway

**DOI:** 10.1038/s41420-022-01224-w

**Published:** 2022-10-28

**Authors:** Kunyu Shen, Jinhua Miao, Qiongdan Gao, Xian Ling, Ye Liang, Qin Zhou, Qirong Song, Yuxin Luo, Qinyu Wu, Weiwei Shen, Xiaonan Wang, Xiaolong Li, Youhua Liu, Shan Zhou, Ying Tang, Lili Zhou

**Affiliations:** 1 Division of Nephrology, Nanfang Hospital, Southern Medical University; National Clinical Research Center for Kidney Disease; State Key Laboratory of Organ Failure Research; Guangdong Provincial Institute of Nephrology; Guangdong Provincial Key Laboratory of Renal Failure Research, Guangzhou, 510515 China; 2 Department of Nephrology, The Third Affiliated Hospital of Southern Medical University, Guangzhou, China

**Keywords:** Acute kidney injury, Autophagy

## Abstract

Acute kidney injury (AKI) is in high prevalence in the world. However, the therapeutic strategies for AKI are still in mystery. Studies have shown to improve autophagy and lysosomal function could inhibit AKI. But their modulators need to be explored in detail. Annexin A2 (ANXA2) is a phospholipid-binding protein involving in organelle membrane integrity function, suggesting its important role in autophagy and lysosome homeostasis. It implicates ANXA2 potentially protects against AKI. However, this has not been elucidated. Herein, we found that ANXA2 is increased in renal tubules in cisplatin-induced AKI mice. Ectopic expression of ANXA2 improved lysosomal functions and enhanced autophagic flux, further protecting against renal tubular cell apoptosis and kidney injury. Conversely, knockdown of ANXA2 inhibited lysosomal function and autophagy, which aggravated the progression of AKI. Transcriptome sequencing revealed β-catenin signaling is highly responsible for this process. In vitro, we found ANXA2 induced β-catenin activation, further triggering T-cell factor-4 (TCF4)-induced transcription factor EB (TFEB). Furthermore, TFEB promoted lysosome biogenesis to enhance autophagic flux, resulting in the alleviation of AKI. Our new findings underline ANXA2 is a new therapeutic potential for AKI through modulating autophagy and lysosomal function. The underlying mechanism is associated with its inductive effects on β-catenin/TFEB pathway.

## Introduction

Acute kidney injury (AKI) is in high prevalence worldwide [1], and is in high rates to progress into chronic kidney disease (CKD), an irreversible process [2]. AKI is characterized by renal tubular epithelial cell apoptosis [3], which is mainly caused by ischemic injury [4]. AKI commonly occurs in hospitalized patients who are suffering cardiac or gastrointestinal surgery [5, 6]. Besides that, AKI is also highly involved in those patients with cancer who are subjects administering chemotherapeutic drugs such as cisplatin [2, 7–9]. Of note, cisplatin is an agent which is metabolized through the kidney [10], and could trigger AKI at high incidence [9, 11]. However, the therapeutic strategies for AKI are still in blank nowadays.

Autophagy is highly conserved during evolution, to maintain physiological homeostasis [12]. It is a series of self-defense process, including phagophore, autophagosome and autolysosome formation, as well as cargo degradation and recycling [13]. Studies have reported that autophagy protects against AKI [14, 15]. However, the modulators still need to be determined. Of interest, the effective membrane biogenesis plays an important role in initiating autophagy [16, 17]. This provides a novel prospect for developing therapeutic strategies for AKI.

Annexin A2 (ANXA2), a 39-kDa phospholipid-binding protein, belongs to the Annexin family [18] and plays a key role in membrane organization, traffic, and fusion [19, 20]. With the high involvement in dynamic events of membrane events [21, 22], ANXA2 plays a crucial role in mediating autophagy [23, 24]. Indeed, ANXA2 ablation leads to the defects of phagophore membrane initiation [25] and the deficiency in autophagy setting [26]. This suggests ANXA2 could protect against AKI. However, it is not clarified although a previous report showed ANXA2 increased in AKI [27].

Wnt/β-catenin signaling is a developmental signal [28]. Previous reports showed Wnt/β-catenin plays an important role in protecting against AKI [29, 30]. Upon Wnt stimulation, β-catenin translocates into nuclei to induce downstream targets through T-cell factor (TCF)/Lymphoid enhancer factor (LEF) transcription factor family [31]. Without Wnt, cytoplasmic β-catenin is commonly degraded by a destruction complex containing glycogen synthase kinase 3β (GSK3β) [32], a key regulator for β-catenin degradation [33]. Interestingly, ANXA2 is a modulator of GSK3β [34] and further activates β-catenin signals in cancer cells [35]. But the relationship between ANXA2 and β-catenin signaling in renal tubular cells has not been clarified.

In this study, we found ANXA2 could promote lysosomal functions and autophagy. The underlying mechanism is related to the activation of β-catenin signaling. Innovatively, we further identified transcription factor EB (TFEB), a key transcription factor of lysosome biogenesis and homeostasis, is a new downstream target of β-catenin. Overall, our study underlies the important role and underlying mechanisms of ANXA2 in AKI. We further provided an important strategy to treat AKI.

## Results

### ANXA2 is upregulated in cisplatin-induced AKI mice

**Fig. 1:**
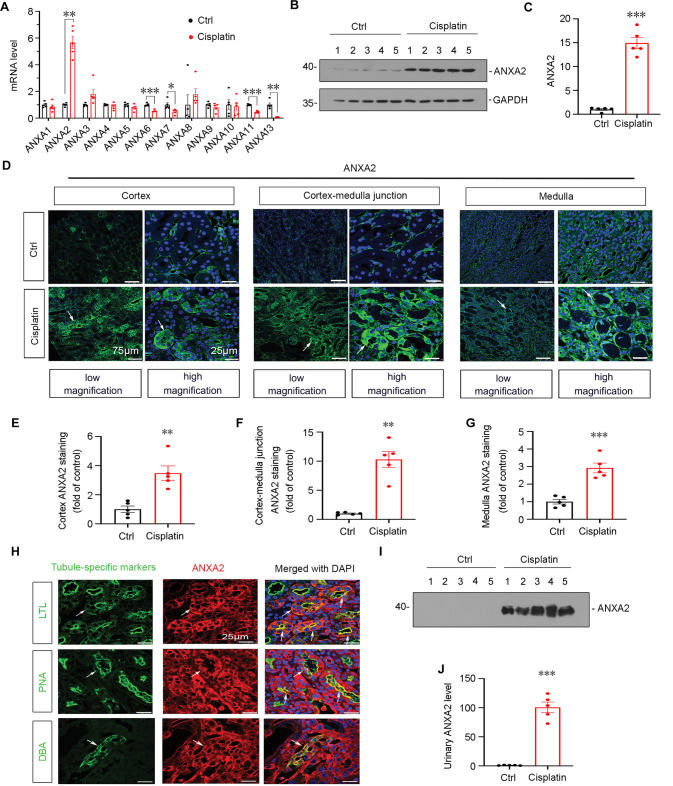
ANXA2 is upregulated in cisplatin-induced AKI mice. **A** Quantitative data shows the relative expression of different ANXA family members in cisplatin-treated mice or control mice. *P < 0.05, **P < 0.01, ***P < 0.001 versus Ctrl group (*n* = 5). **B**, **C** Western blot analysis shows the expression of ANXA2 in two groups. ***P < 0.001 versus Ctrl (*n* = 5). **D** Immunofluorescence staining demonstrates the expression of ANXA2 is upregulated across different sections in AKI-affected kidney. White arrows indicate positive staining. Scale bars, 75 μm (low magnification); 25 μm (high magnification). **E**–**G** Quantification of positive staining. **P < 0.01, ***P < 0.001 versus Ctrl group (*n* = 5). **H** Co-localization of ANXA2 (red) with LTL (green, a marker for proximal renal tubule), PNA (green, a marker for distal renal tubule) and DBA (green, a marker for collecting duct) are shown. Results show ANXA2 is largely expressed in proximal and distal tubules, but localizes in collecting ducts to a less extent. White arrows indicate positive staining. Scale bars, 25 μm. **I, J** Western blotting shows urinary ANXA2 expression. Mouse urine samples were collected and tested by western blotting. The urinary ANXA2 expression was quantified by normalizing to Urinary creatinine. ***P < 0.001 versus Ctrl (*n* = 5). Abbreviations: ANXA2 Annexin A2, Ctrl control.

The gene expression of annexins family was first identified in cisplatin-treated mice. As shown, ANXA2 increased the most in kidneys after cisplatin treatment (Fig. 1A). We next assessed the expression of ANXA2 protein. As shown, it is also increased (Fig. 1B, C). Immunofluorescence staining also revealed ANXA2 increased in cisplatin-treated kidneys across different areas, including the cortex, cortex-medulla junction and medulla (Fig. 1D–G). We further performed co-staining of ANXA2 with different markers specifically indicating segmental tubules. As shown, ANXA2 was primarily co-localized with LTL and PNA, the proximal and distal tubule markers, and to a less extent, co-localized with DBA, a marker for collecting ducts (Fig. 1H). We also tested the expression of ANXA2 in urine from cisplatin-treated mice, and found it was strikingly increased in AKI model (Fig. 1I, J).

### ANXA2 protects against AKI

**Fig. 2:**
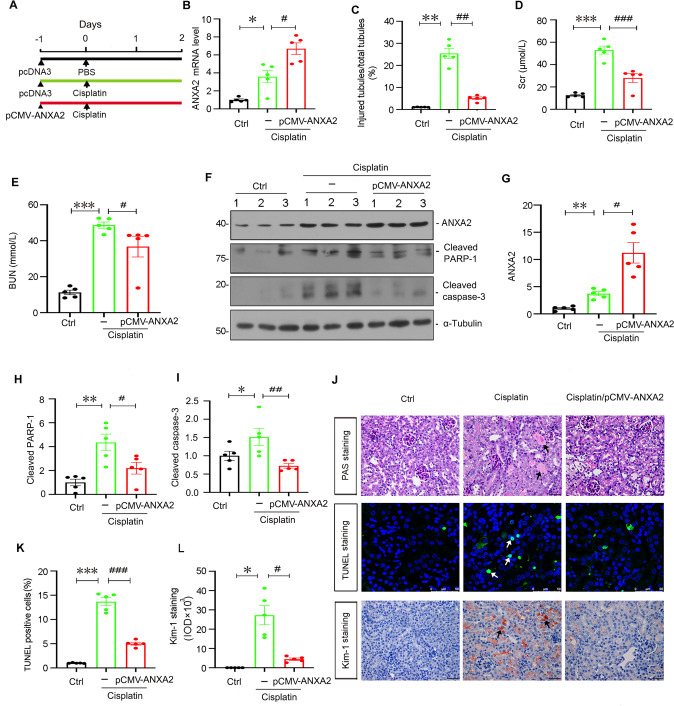
ANXA2 protects against cisplatin-induced AKI. **A** The experimental design is shown. **B** Quantitative PCR shows ANXA2 is upregulated by injection of an expression plasmid. *P < 0.05 versus Ctrl (*n* = 5); ^#^*P* < 0.05 versus cisplatin alone (*n* = 5). **C** Quantitations of tubular injury in 3 groups are shown. **P < 0.01 versus Ctrl (*n* = 5); ^##^*P* < 0.01 versus cisplatin alone (*n* = 5). **D**, **E** Quantitation of Scr and BUN levels in 3 groups are shown. ***P < 0.001 versus Ctrl (*n* = 5); ^#^*P* < 0.05; ^###^*P* < 0.001 versus cisplatin alone (*n* = 5). Representative western blotting (**F**) and quantitative data showing the expression of ANXA2 (**G**), cleaved PARP-1 (**H**) and cleaved caspase-3 (**I**). *P < 0.05, **P < 0.01 versus Ctrl (*n* = 5); ^#^*P* < 0.05, ^##^*P* < 0.01 versus cisplatin alone (*n* = 5). (**J**) Representative photographs show PAS, TUNEL, and Kim-1 staining in 3 groups. Black and white arrows indicate positive staining. Scale bar, 50 μm. **K**, **L** Quantitative data show the difference of TUNEL-positive tubular cell numbers and positive staining of Kim-1 in 3 groups. *P < 0.05, ***P < 0.001 versus Ctrl (*n* = 5); ^#^*P* < 0.05, ^###^*P* < 0.001 versus cisplatin alone (*n* = 5). Abbreviations: ANXA2 annexin A2, Ctrl control, pcDNA3 pcDNA3 plasmid.

We then examined whether ANXA2 protects against cisplatin-induced AKI. The experimental design was shown in Fig. 2A. ANXA2 was overexpressed by injection of an expression plasmid (pCMV-ANXA2) through a hydrodynamic approach as previously reported [36]. The efficacy of ANXA2 overexpression was demonstrated by qRT-PCR (Fig. 2B). We next tested whether ANXA2 inhibited tubular cell injury and preserved kidney function. As shown, ectopic ANXA2 significantly retarded tubular injury (Fig. 2C), and inhibited the increase in serum creatinine and urea nitrogen levels (Fig. 2D, E). We next measured cell apoptosis. As shown, in cisplatin-treated mice, ectopic ANXA2 further induced the upregulation of ANXA2 (Fig. 2F, G), which significantly inhibited the increase in cleaved PARP-1 and caspase-3 expression, the key executors for apoptosis (Fig. 2F, H, I). We next performed PAS and TUNEL staining. As shown, cisplatin treatment induced dilated tubules with hyaline casts and cell detachment, which was blocked by co-treatment with ectopic ANXA2 (Fig. 2J). Consistently, TUNEL staining also indicated ANXA2 significantly blocked cell apoptosis (Fig. 2J, K). The similar result was observed when kim-1, a tubular cell injury marker, was examined by immunohistochemistry (Fig. 2J, L).

### ANXA2 induces autophagy and lysosomal functions, and is associated with Wnt/β-catenin signaling

**Fig. 3:**
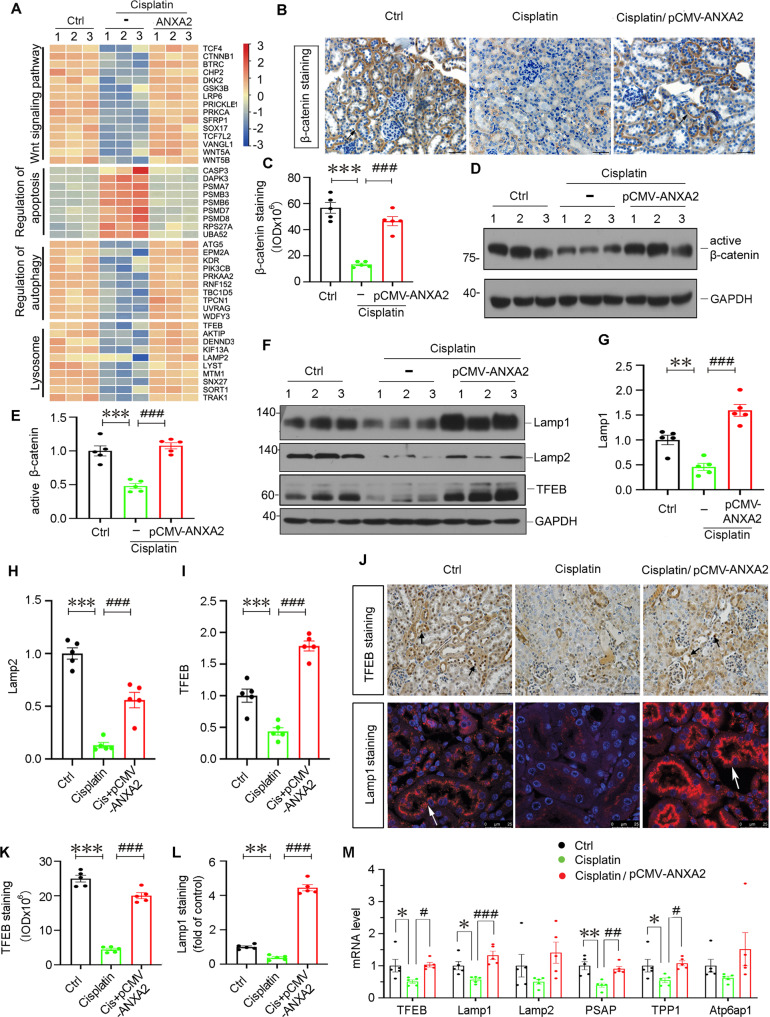
ANXA2 induces autophagy and lysosomal functions, and is associated with Wnt/β-catenin signaling. **A** Heatmap shows the FPKM value of genes in different groups. Relevant genes are categorized into different GSEA gene sets. The color ranges from red to blue represents the log2-ranked FPKM value from large to small. Representative photographs show β-catenin expression (**B**) and its quantitation (**C**) in 3 groups. Black arrows indicate positive staining. Scale bars, 50 μm. ***P < 0.001 versus Ctrl (*n* = 5); ^###^*P* < 0.001 versus cisplatin alone (*n* = 5). Representative western blotting (**D**) and quantitative data (**E**) show ANXA2 induces β-catenin activation. Numbers (1–3) indicate individual animals in each group. ***P < 0.001 versus Ctrl (*n* = 5); ^###^*P* < 0.001 versus cisplatin alone. Representative western blotting (**F**) and quantitative data (**G**–**I**) show the expression of Lamp1 (**G**), Lamp2 (**H**) and TFEB (**I**) in three groups. Numbers (1–3) indicate individual animals in each group. **P < 0.01, ***P < 0.001 versus Ctrl (*n* = 5); ^###^*P* < 0.001 versus cisplatin alone (*n* = 5). **J** Representative photographs show TFEB (the top panel) and Lamp1 (the bottom panel) expression in 3 groups. Arrows indicate positive staining. Scale bars, 50 μm or 25 μm. **K**, **L** Quantification of TFEB and Lamp1 staining are shown. **P < 0.01, ***P < 0.001 versus Ctrl (*n* = 5); ^###^*P* < 0.001 versus cisplatin alone (*n* = 5). **M** qPCR results show mRNA levels of TFEB, Lamp1, Lamp2, PSAP, TPP1 and Atp6ap1 in different groups. *P < 0.05, **P < 0.01 versus Ctrl (*n* = 5); ^#^*P* < 0.05, ^##^*P* < 0.01, ^###^*P* < 0.001 versus cisplatin alone (*n* = 5). Abbreviations: ANXA2 annexin A2, Ctrl control, FPKM fragments per kilobase of transcript per million, GSEA Gene Set Enrichment Analysis, pcDNA3 empty vector.

We then performed RNA sequencing in 3 groups of mice. The heatmap and GSEA analysis demonstrated differential gene expression profile in regulation of apoptosis and autophagy, lysosome function. Interestingly, we also observed Wnt/β-catenin signaling was induced by ANXA2 overexpression (Fig. 3A and Supplemental Fig. S1).

We then checked β-catenin expression. As shown in Fig. 3B–E, β-catenin expression and its activation were suppressed by cisplatin treatment, but significantly restored by ectopic expression of ANXA2. We then assessed lysosomal functions. As shown, the expressional levels of Lamp1, Lamp2 and TFEB, the lysosome markers, were all downregulated in cisplatin-treated mice, but significantly restored in ANXA2-overexpressed mice (Fig. 3F–I). The similar results were also observed when TFEB and Lamp1 were tested by immunostaining (Fig. 3J–L). We also performed multiple qPCR analysis to assess lysosome-related genes including TFEB, Lamp1, Lamp2, PSAP, TPP1, and Atp6ap1. As shown, they were downregulated by cisplatin treatment, but significantly restored by co-treatment with ANXA2 overexpression (Fig. 3M).

### ANXA2 promotes lysosomal biogenesis and enhances autophagic flux in cisplatin-treated mice

**Fig. 4:**
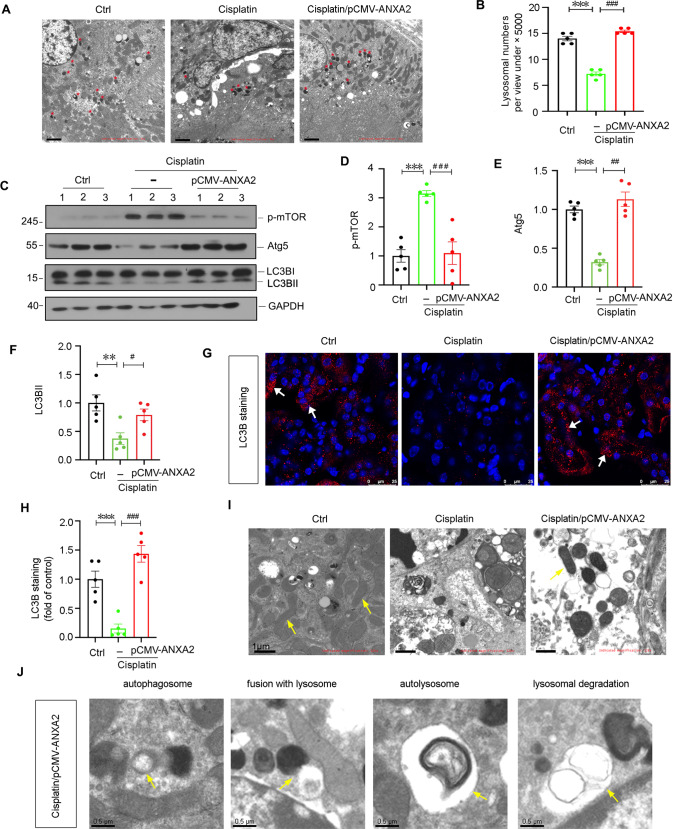
ANXA2 promotes lysosome-mediated autophagy function in cisplatin-treated mice. **A** Representative TEM photographs show lysosomes (red stars) in renal tubular cells. Scale bar, 2 μm. **B** The lysosomal numbers in 3 groups were quantified. Randomly selected 10 cells from each group were photographed under the view of ×5000 magnification. ***P < 0.001 versus Ctrl (*n* = 5); ^###^*P* < 0.001 versus cisplatin alone (*n* = 5). **C**–**F** Representative western blotting and quantification show the expression of p-mTOR (**D**), Atg5 (**E**) and LC3BII (**F**) in 3 groups. **P < 0.01, ***P < 0.001 versus Ctrl (*n* = 5); ^#^*P* < 0.05, ^##^*P* < 0.01, ^###^*P* < 0.001 versus cisplatin alone (*n* = 5). Representative photographs show immunofluorescence staining of LC3B (**G**) and its quantification is shown (**H**). Scale bars, 25 μm. White arrows indicate positive LC3B puncta. ***P < 0.001 versus Ctrl (*n* = 5); ^###^*P* < 0.001 versus cisplatin alone (*n* = 5). **I** Representative TEM photos show mitochondria in 3 groups. Yellow arrow indicates mitochondria. Scale bar, 1 μm. **J** Representative TEM photos show a series of processes of autophagic flux in the group co-treatment with cisplatin and ANXA2. Yellow arrows indicate a double-membrane-shaped autophagosome; an autophagosome which is fusing with a lysosome; an autolysosome and an autophagolysosomal vacuole undergoing degradation, respectively. Scale bars, 0.5 μm. Abbreviations: ANXA2 annexin A2, Ctrl control, pcDNA3 empty vector.

We then assessed the lysosome biogenesis in proximal tubular cells. Through electron microscopy (TEM) analysis (Fig. 4A, B), we found cisplatin treatment decreased the numbers of lysosomes, but this was blocked by ectopic expression of ANXA2. As lysosome biogenesis facilitates autophagy function. We then assessed autophagy in 3 groups of mice. As shown (Fig. 4C–F), ANXA2 overexpression significantly attenuated p-mTOR expression, and increased the expression of Atg5 and LC3BII. Immunofluorescence staining of LC3B further revealed ectopic expression of ANXA2 greatly promoted the maturation of LC3B, as characterized by the assembly of positive puncta (Fig. 4G, H). We also found ANXA2 preserved the healthy mitochondria, and effectively triggered a series of autophagic processes including autophagosome formation, autophagosome fusion with lysosome, autolysosome formation, as well as lysosome-mediated degradation (Fig. 4I, J).

### Knockdown of ANXA2 worsens cisplatin-induced AKI in vivo

**Fig. 5:**
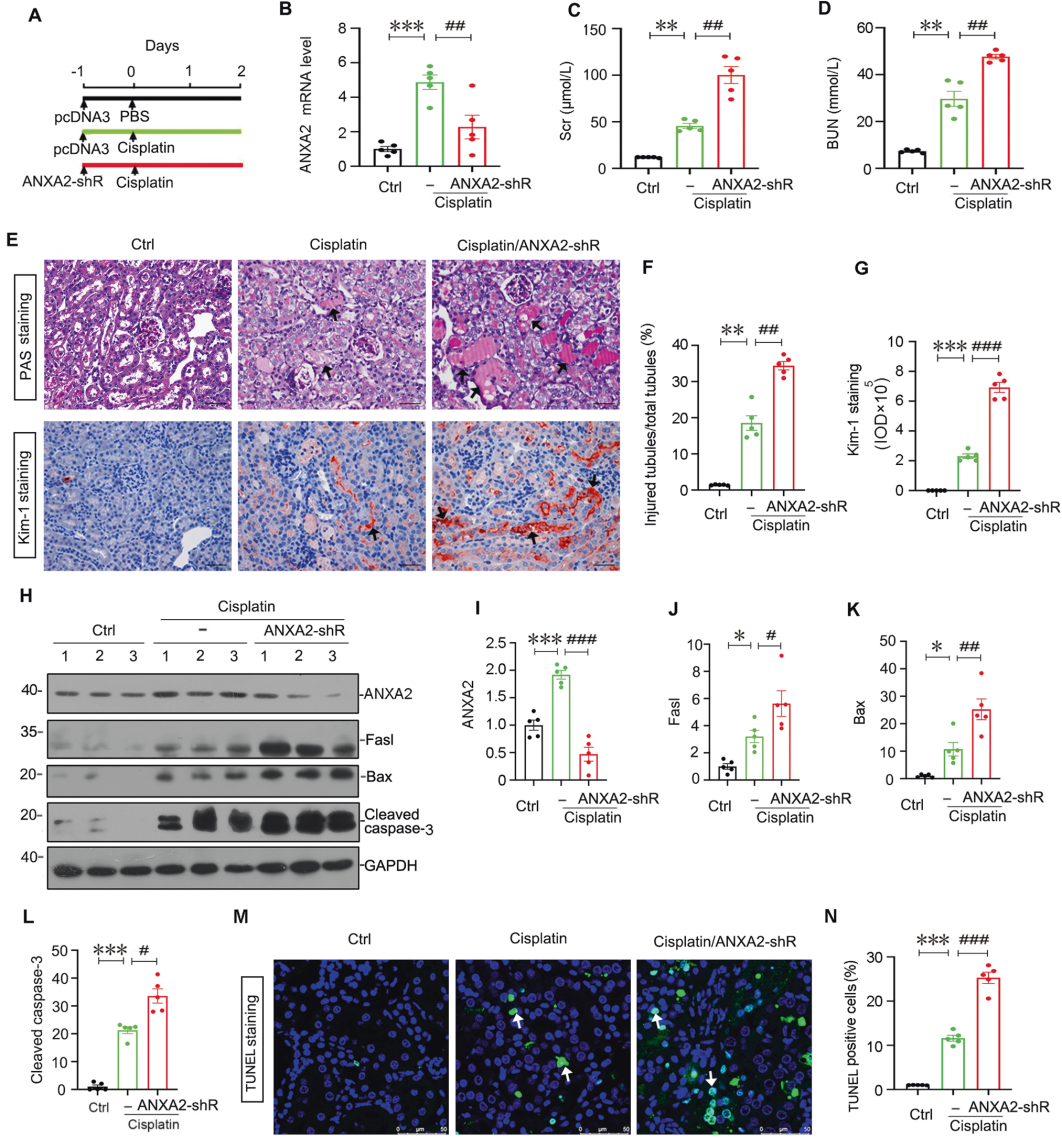
Knockdown of ANXA2 worsens AKI in vivo. **A** The schematic graph shows the experimental design. **B** The quantitative data shows the mRNA levels of ANXA2. ***P < 0.001 versus Ctrl (*n* = 5); ^##^*P* < 0.01 versus cisplatin alone (*n* = 5). The quantifications of Scr (**C**) and BUN (**D**) levels in 3 groups are shown. **P < 0.01 versus Ctrl (*n* = 5); ^##^*P* < 0.01 versus cisplatin alone (*n* = 5). **E**–**G** Representative photographs show PAS and Kim-1 staining and their quantifications. Scale bars, 50 μm. In PAS staining, arrows indicate severely injured renal tubules with naked brush boarder; In Kim-1 staining, arrows indicate positive staining. Scale bar, 50 μm. **P < 0.01, ***P < 0.001 versus Ctrl (*n* = 5); ^##^*P* < 0.01, ^###^*P* < 0.001 versus cisplatin alone (*n* = 5). Representative western blotting (**H**) and quantifications show the expression of ANXA2 (**I**), Fasl (**J**), Bax (**K**) and cleaved caspase-3 (**L**).*P < 0.05, ***P < 0.001 versus Ctrl (*n* = 5); ^#^*P* < 0.05, ^##^*P* < 0.01, ^###^*P* < 0.001 versus cisplatin alone (*n* = 5). Representative photos show TUNEL-positive cells (**M**) and its quantification (**N**). Scale bar, 50 μm. ***P < 0.001 versus Ctrl (*n* = 5); ^###^*P* < 0.001 versus cisplatin alone (*n* = 5). Abbreviations: ANXA2 annexin A2, Ctrl control, pcDNA3 empty vector; shRNA small hairpin RNA.

We then knocked down ANXA2 through shRNA-mediated approach in cisplatin-treated mice (Fig. 5A). The efficacy of ANXA2 knockdown was confirmed by qPCR (Fig. 5B). As shown, ANXA2 knockdown further aggravated the increase in Scr and BUN levels in cisplatin-treated mice (Fig. 5C, D). Furthermore, PAS and Kim-1 staining revealed ANXA2 knockdown exacerbated cell injury in renal tubular cells (Fig. 5E–G). We further measured cell apoptosis. As shown, knockdown of ANXA2 further increased the upregulation of apoptosis-related proteins such as Fasl, Bax, and cleaved caspase-3 in cisplatin-treated mice (Fig. 5H–L). Consistently, TUNEL staining also revealed interference of ANXA2 further induced cell apoptosis in cisplatin-treated mice (Fig. 5M, N).

### Knockdown of ANXA2 blocked lysosomal function and inhibited autophagy in cisplatin-induced AKI mice through inhibiting β-catenin signaling

**Fig. 6:**
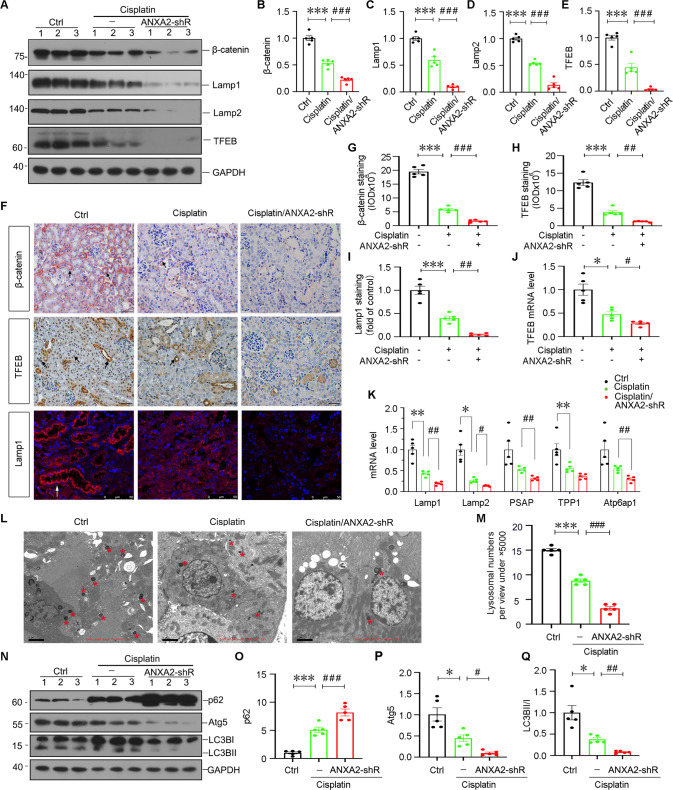
Knockdown of ANXA2 decreases lysosomal function and inhibited autophagy. Representative western blotting (**A**) and quantification of β-catenin (**B**), Lamp1 (**C**), Lamp2 (**D**) and TFEB (**E**) are shown. ***P < 0.001 versus Ctrl (*n* = 5); ^###^*P* < 0.001 versus cisplatin alone (*n* = 5). **F** Representative photographs show β-catenin, TFEB and Lamp1 staining in 3 groups. Arrows indicate positive staining. Scale bar, 50 μm. Quantification of β-catenin (**G**), TFEB (**H**) and Lamp1 (**I**) staining are shown. ***P < 0.001 versus Ctrl (*n* = 5); ^##^*P* < 0.01, ^###^*P* < 0.001 versus cisplatin alone (*n* = 5). qPCR results show the mRNA levels of TFEB (**J**), Lamp1, Lamp2, PSAP, TPP1 and Atp6ap1 (**K**). *P < 0.05, **P < 0.01 versus Ctrl (*n* = 5); ^#^*P* < 0.05, ^##^*P* < 0.01 versus cisplatin alone (*n* = 5). **L**, **M** Representative TEM photos show lysosomes (red stars) in 3 groups and the quantifications are shown. Scale bar, 2 μm. Randomly selected 10 cells from each group were photographed and quantified under the view of ×5000 magnification. ***P < 0.001 versus Ctrl (*n* = 5); ^###^*P* < 0.001 versus cisplatin alone (*n* = 5). Representative western blotting (**N**) and the quantification show the expression of p62 (**O**), Atg5 (**P**) and LC3BII/I (**Q**). *P < 0.05, ***P < 0.001 versus Ctrl (*n* = 5); ^#^*P* < 0.05, ^##^*P* < 0.01, ^###^*P* < 0.001 versus cisplatin alone (*n* = 5). Abbreviations: ANXA2 annexin A2, Ctrl control, pcDNA3 empty vector, shRNA, small hairpin RNA.

We then assessed the expression of β-catenin. As shown, cisplatin treatment decreased the expression of β-catenin, but knockdown of ANXA2 further inhibited its expression (Fig. 6A, B). We further assessed lysosome and autophagy function. Western blotting analysis demonstrated ANXA2 knockdown further aggravated the decrease in lysosome-related proteins such as Lamp1, Lamp2, and TFEB (Fig. 6A, C–E). The similar results were also observed when β-catenin, TFEB, and Lamp1 were assessed by immunostaining (Fig. 6F–I). We also tested the expression of TFEB and other lysosome-related genes by qPCR analysis (Fig. 6J, K), and found they were further downregulated by knockdown of ANXA2. We then observed the lysosomes directly by TEM analysis. As shown in Fig. 6L, M, cisplatin treatment decreased the lysosome numbers, but it was further exacerbated by knockdown of ANXA2. Autophagy function was further assessed. As shown (Fig. 6N–Q), cisplatin treatment induced the increase in p62, and decrease in Atg5 and LC3BI to II transformation, but this was further aggravated by ANXA2 knockdown.

### ANXA2 inhibits cell apoptosis and promotes autophagy in cultured renal tubular cells

**Fig. 7:**
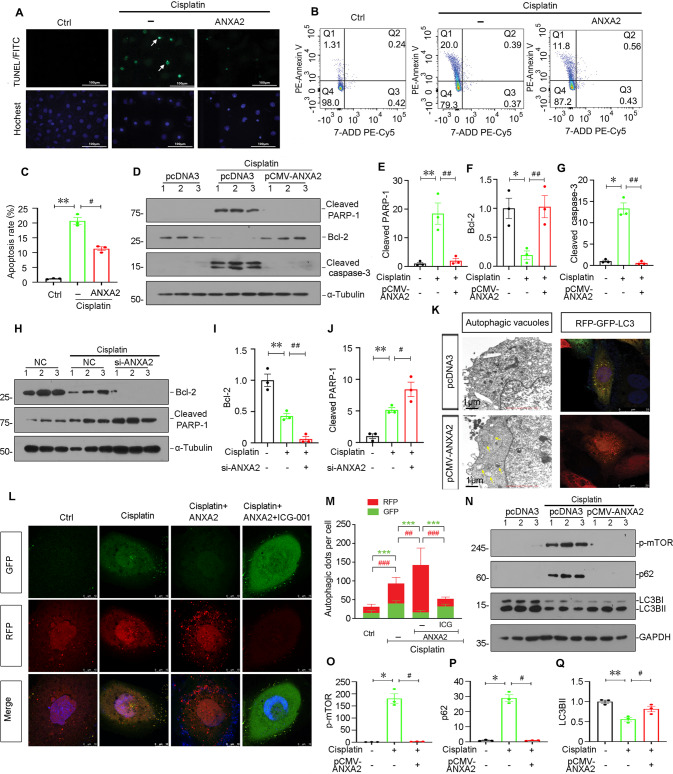
ANXA2 blocks renal tubular cell apoptosis and promotes autophagy in vitro. **A** Representative TUNEL staining shows cell apoptosis (green fluorescence). Scale bar, 100 μm. HK-2 cells were stimulated by cisplatin (25 μg/mL) for 24 h. Some cells were pretreated with recombinant human ANXA2 protein (2 μg/mL) for 24 h. **B**, **C** Flow cytometry analysis showing the ratio of apoptotic cells (annexin V5 positive/7-ADD negative). **P < 0.01 versus Ctrl (*n* = 3); ^#^*P* < 0.05 versus Cisplatin alone (*n* = 3). Representative western blotting (**D**) and quantitative data of the protein expression of cleaved PARP-1 (**E**), Bcl-2 (**F**) and cleaved caspase-3 (**G**) in different groups are shown. HK-2 cells were transfected with ANXA2 expression plasmid for 6 h, and then treated with cisplatin (25 μg/mL) for 24 h. *P < 0.05, **P < 0.01 versus Ctrl (*n* = 3); ^##^*P* < 0.01 versus Cisplatin alone (*n* = 3). Representative western blotting (**H**) and quantitative data of the expression of Bcl-2 (**I**) and cleaved PARP-1 (**J**) in different groups are shown. HK-2 cells were transfected with si-ANXA2 for 24 h, and then treated with cisplatin (25 μg/mL) for 24 h. **P < 0.01 versus Ctrl (*n* = 3); ^#^*P* < 0.05, ^##^*P* < 0.01 versus Cisplatin alone (*n* = 3). **K** Representative TEM photographs show ANXA2 increased the numbers of autophagic vacuoles (left panel). Arrows indicate autophagic vacuoles. Scale bar, 1 μm; Representative images show ANXA2 promotes autophagic flux as evidenced by enhanced RFP (red) positive LC3 puncta (right panel). Scale bar, 25 μm. **L** ANXA2 promotes autophagic flux in cisplatin-treated HK-2 cells. HK-2 cells were transfected with RFP-GFP-LC3 lentivirus for 24 h, and then treated with ANXA2 recombinant protein (2 μg/mL), or ANXA2 with cisplatin (25 μg/mL) for another 24 h. Some cells were also co-treated with ICG-001 (10 μM). Red fluorescence shows acidic-pH, and LC3B-positive autolysosomes; Green fluorescence shows neutral-pH LC3B-positive autophagosomes. Scale bars, 10 μm. **M** Quantification of autophagosomes (green fluorescence) and autolysosomes (red fluorescence) numbers. Randomly selected 10 cells were calculated for each group. Quantitative data of autophagosomes (green) and autolysosomes (red) are shown in the bar graph. ***P < 0.001, ^##^*P* < 0.01, ^###^*P* < 0.001 versus the indicated group (*n* = 10). Representative western blotting (**N**) and quantitative data show the expression of p-mTOR (**O**), p62 (**P**) and LC3BII (**Q**) in different groups. *P < 0.05, **P < 0.01 versus Ctrl (*n* = 3); ^#^*P* < 0.05 versus Cisplatin alone (*n* = 3). Abbreviations: ANXA2 annexin A2, Ctrl control, ICG ICG-001, pcDNA3 empty vector, TUNEL terminal deoxynucleotidyl transferase dUTP nick-end labeling.

HK-2 cells, a human proximal tubular cell line, were also cultured. Firstly, cell apoptosis was assessed by TUNEL and Annexin V staining. As shown, cisplatin treatment induced cell apoptosis in HK-2 cells, but this was blocked by co-treatment with ANXA2 (Fig. 7A–C). Western blotting analysis also revealed ANXA2 inhibited the expression of apoptosis-related proteins including cleaved PARP-1 and caspase-3, and restored the expression of Bcl-2, an anti-apoptotic protein (Fig. 7D–G). We further transfected HK-2 cells with siRNA to ANXA2 to knockdown ANXA2. As shown, cisplatin treatment decreased the expression of Bcl-2 expression and increased cleaved PARP-1 expression, but this was further aggravated by ANXA2 knockdown in cisplatin-treated cells (Fig. 7H–J).

We next assessed autophagy. HK-2 cells were firstly transfected with ANXA2 expressing plasmid. As shown, overexpression of ANXA2 increased the numbers of autophagic vacuoles through the assessment by TEM (Fig. 7K). Autophagic flux was also assessed. HK-2 cells were transfected with a lentiviral biosensor containing GFP-LC3B and RFP-LC3B, which enables LC3B-positive, neutral-pH autophagosomes as green fluorescence, whereas LC3B-positive acidic-pH autophagolysosomes exhibit red fluorescence. As shown (Fig. 7K), overexpression of ANXA2-induced autophagolysosome formation. We also examined autophagic flux in cisplatin-treated cells. As shown (Fig. 7L, M), the fluorescent colors of LC3B-positive puncta in cisplatin-treated cells were mostly green and yellow (overlap of red and green fluorescence). This suggested cisplatin induced autophagosome accumulation without fusion with lysosomes. However, a strong staining of RFP-LC3B-positive puncta was observed in ANXA2 co-treated group, suggesting the important role of ANXA2 in enhancing autophagic flux. However, this was blocked by ICG-001, an inhibitor of β-catenin-activated transcription, further demonstrating β-catenin meditates ANXA2-induced autophagic pathway. We also assessed the expression of autophagy-related proteins by western blotting. As shown, cisplatin-induced increase in p-mTOR and p62, and decrease in LC3BII formation was blocked by overexpression of ANXA2 (Fig. 7N–Q).

### ANXA2 promotes lysosomal functions in cultured renal tubular cells

**Fig. 8:**
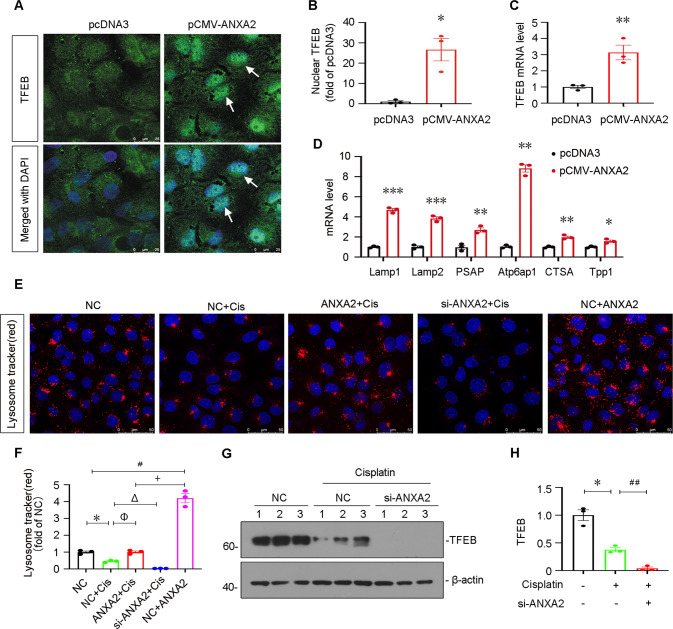
ANXA2 promotes lysosomal functions through β-catenin/TCF4 signaling. **A** Representative photographs show the immunofluorescence staining of TFEB (**B**) and its quantification. White arrows indicate nuclear staining of TFEB. Scale bar, 25 μm. *P < 0.05 versus pcDNA3 (*n* = 3). **C** qPCR shows the mRNA levels of TFEB. **P < 0.001 versus pcDNA3 (*n* = 3). **D** qPCR results show the mRNA levels of lysosome-related genes. HK-2 cells were transfected with ANXA2 expression plasmid for 24 h. *P < 0.05, **P < 0.01, ***P < 0.001 versus pcDNA3 (*n* = 3). **E**, **F** Representative photographs and quantification show lysosome staining in different groups. Scale bar, 50 μm. HK-2 cells were pretreated with recombinant ANXA2 protein (2 μg/mL) or transfected with siRNA to ANXA2 or negative control (NC) for 24 h, and then treated with cisplatin (25 μg/mL) or medium alone for another 24 h. *P < 0.05, cisplatin (*n* = 3) versus NC alone (*n* = 3); ^#^*P* < 0.05, ANXA2 (*n* = 3) versus NC alone (*n* = 3); ^+^*P* < 0.05, cisplatin+ANXA2 (*n* = 3) versus ANXA2 + NC (*n* = 3); ^φ^*P* < 0.05, cisplatin+ANXA2 (*n* = 3) versus cisplatin+NC (*n* = 3); ^▵^*P* < 0.05, cisplatin+si-ANXA2 (*n* = 3) versus cisplatin+NC (*n* = 3). Representative western blotting (**G**) and quantitative data show the expression of TFEB (**H**) in different groups. *P < 0.05 versus Ctrl (*n* = 3); ^##^*P* < 0.01 versus Cisplatin alone (*n* = 3). HK-2 cells were transfected with si-ANXA2 for 24 h, and then treated with cisplatin (25 μg/mL) for 24 h. Abbreviations: TFEB transcription factor EB, Lamp1 lysosomal associated membrane protein 1, TPP1 tripeptidyl peptidase 1.

We further assessed lysosomal functions in vitro. In cultured HK-2 cells, overexpression of ANXA2 induced the nuclear translocation of TFEB (Fig. 8A, B). TFEB mRNA and other lysosome-related gene expression were further assessed by qRT-PCR. As shown, they were all significantly upregulated by overexpression of ANXA2 (Fig. 8C, D). Furthermore, lysosome biogenesis was also examined by a lysosomal tracker probe staining. As shown, cisplatin treatment decreased the lysosome numbers, but this was blocked by overexpression of ANXA2 or aggravated by knockdown of ANXA2 (Fig. 8E, F). We next assessed the effects of ANXA2 knockdown in TFEB protein expression. As shown, cisplatin treatment decreased the expression of TFEB, but this was exacerbated after knockdown of ANXA2 (Fig. 8G, H).

### ANXA2 induces TFEB transcription and activation through activating β-catenin

**Fig. 9:**
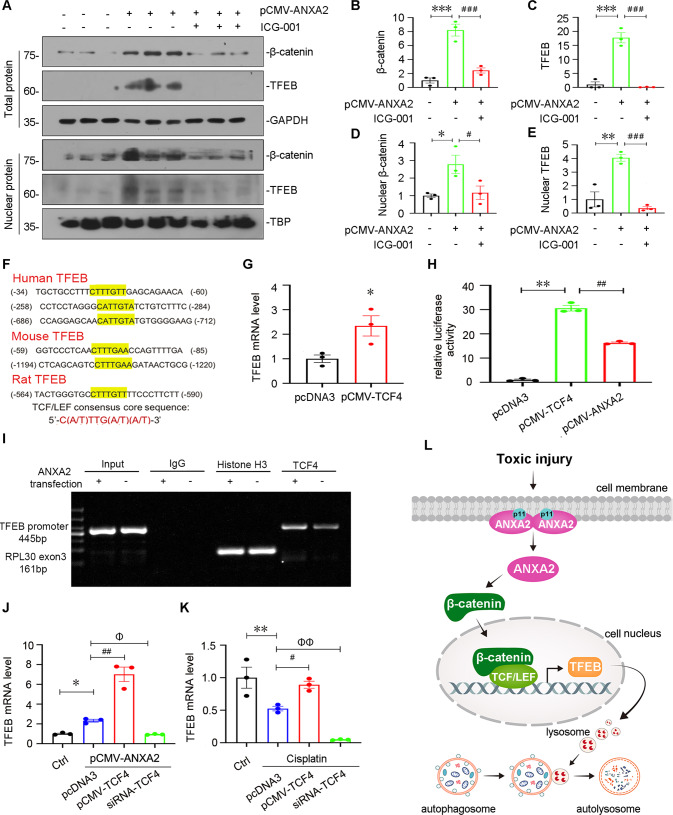
ANXA2 promotes lysosomal functions through β-catenin/TCF4/TFEB signaling. Representative western blotting (**A**) and quantitative data of the expression of β-catenin (**B**) and TFEB (**C**) in total protein, β-catenin (**D**) and TFEB (**E**) in nuclear protein are shown. *P < 0.05, **P < 0.01, ***P < 0.001 versus pcDNA3 (*n* = 3); ^#^*P* < 0.05, ^###^*P* < 0.001 versus pCMV-ANXA2 alone (*n* = 3). HK-2 cells were transfected with ANXA2 expression plasmid for 6 h, and then treated with ICG-001 (10 μm) for 24 h. **F** Bioinformatics assay showing the potential binding sites of TCF/LEF consensus in TFEB promoter region. **G** qPCR results showing the mRNA level of TFEB in 2 groups. *P < 0.05 versus pcDNA3 (*n* = 3). **H** Luciferase assay shows the transcriptional activity of TFEB. HK-2 cells were transfected with TCF4 or ANXA2 expression plasmid or pcDNA3, and co-transfected with pGL3-TFEB reporter plasmid. The transcriptional activity of TFEB was detected by the relative activity of luciferase. ^**^P < 0.01, pCMV-TCF4 (*n* = 3) versus ctrl (*n* = 3); ^##^*P* < 0.01, pCMV-ANXA2 (*n* = 3) versus pCMV-TCF4 (*n* = 3). **I** Representative ChIP-PCR analysis shows the binding of TCF4 antibody to TFEB promoter. HK-2 cells were transfected with ANXA2 expression plasmid or pcDNA3 for 24 h. Cell lysates were precipitated with an antibody against TCF4, histone H3, or nonimmune IgG, and the ChIP assay was performed for TFEB gene promoter. Total diluted lysate was used as total genomic input DNA. **J** qPCR results showing the mRNA level of TFEB in different groups. HK-2 cells were transfected with ANXA2 expression plasmid or co-transfected with TCF4 expression plasmid or siRNA to TCF4. *P < 0.05 versus ctrl (n = 3); ^φ^*P* < 0.05, ^##^*P* < 0.01 versus pCMV-ANXA2 (*n* = 3). **K** qPCR results showing the mRNA level of TFEB in different groups. HK-2 cells were treated with cisplatin (25 μg/mL) for 24 h, or co-transfected with TCF4 expression plasmid or siRNA to TCF4. The mRNA level of TFEB in different group was assessed by qPCR. **P < 0.01 versus ctrl (*n* = 3); ^#^*P* < 0.05, ^φφ^*P* < 0.01 versus cisplatin alone (*n* = 3). **L** Diagram depicts the potential mechanism of ANXA2 action. As shown, ANXA2 plays a key role in lysosomal functions through β-catenin/TFEB pathway. Specifically, ANXA2 induces nuclear translocation of β-catenin. This leads to β-catenin to trigger TCF4-induced TFEB transcription. Consequently, lysosome biogenesis and lysosome-mediated autophagic flux are promoted. As a result, renal tubular cell apoptosis was inhibited. Abbreviations: ANXA2 annexin A2, ChIP chromatin immunoprecipitation, Ctrl control, TCF4 T-cell factor-4, pcDNA3 empty vector.

We next explored the mediating role of β-catenin in ANXA2 pathway. HK-2 cells were pretreated with ICG-001, and then transfected with ANXA2 expressing plasmid. The expressional levels of β-catenin and TFEB were assessed in whole cell lysis (total protein). As shown, they were upregulated by ANXA2 overexpression, but were blocked by co-treatment with ICG-001. The nuclear translocation of β-catenin represents the active form of it, so as to TFEB. Hence, we further isolated the nuclear fraction (nuclear protein). As shown, overexpression of ANXA2 induced the increase in β-catenin and TFEB expression in nucleus fractions, but this was blocked by co-treatment with ICG-001 (Fig. 9A–E).

We then performed the bioinformatics analysis, and found there are TCF/LEF consensus core sequences in TFEB gene promoter regions. In addition, these biding sites are conserved in human, mouse, and rat (Fig. 9F). HK-2 cells were then transfected with a plasmid expressing TCF4. As shown, overexpression of TCF4 triggered the upregulation of TFEB mRNA levels (Fig. 9G). To further identify the induction of TFEB by TCF4, we then performed luciferase reporter assay. As shown in Fig. 9H, overexpression of ANXA2 induced transcriptional activity of TFEB, and overexpression of TCF4 induced a stronger induction on TFEB transcription. To further identify the direct biding of TCF4 and TFEB induced by ANXA2, we performed the Chromatin Immunoprecipitation (ChIP)-PCR analysis. As shown, overexpression of ANXA2 enhanced the binding of TCF4 transcription factor with TFEB gene promoter (Fig. 9I). To further identify the mediating role of TCF4 in ANXA2-induced TFEB pathway, we transfected HK-2 cells with ANXA2 expression plasmid and TCF4 expressing plasmid or siRNA to TCF4. As shown in Fig. 9J, overexpression of ANXA2 induced the upregulation of TFEB mRNA, and this was further elevated by co-expression of TCF4, but was blocked by interference of TCF4. We further examined the mediating role of TCF4 in cisplatin-treated HK-2 cells. As shown, cisplatin treatment decreased TFEB mRNA levels, but this was blocked by TCF4 overexpression or aggravated by interference of TCF4 (Fig. 9K).

Hence, the schematic diagram demonstrates ANXA2 promotes lysosomal function to reinforce autophagy activity (Fig. 9L). As shown, ANXA2 promotes β-catenin activation, further inducing TFEB transcription. As a result, TFEB further induces lysosome biogenesis and promotes lysosome-mediated autophagic flux. This leads to the blockade of tubular cell apoptosis and the progression of AKI.

## Discussion

The therapeutic strategies of AKI are scarce. To improve autophagy and lysosomal functions could protect against AKI. However, their modulators are still unclear. Annexin family proteins are highly related with the dynamic processes of membrane biogenesis, fusion and separation, suggesting they could participate in the modulation of autophagy and lysosomal function [19, 20]. Interestingly, a previous report showed ANXA2 increases in AKI [27]. However, the authentic role of ANXA2 in AKI remains unclear.

Autophagy defects in renal tubules are associated with AKI [37]. Of note, Autophagy is highly dependent on organelle membrane stabilization and integrity [38]. ANXA2 plays key a role in regulating biological membrane dynamic processes [21] such as autophagy [26]. However, the role of ANXA2 in AKI has not been elucidated. Here, we observed ANXA2 contributes to the integrity of the autophagosome membrane and the fusion of autophagosome with lysosome. Besides that, we also found ANXA2 triggers β-catenin/TCF4 pathway to induce TFEB.

Lysosome stability plays a protective role in autophagic processes [39]. TFEB is a crucial transcription factor in regulating lysosomal biogenesis and functions [40, 41]. We found ANXA2 triggers TFEB transcription to stabilize lysosomal function and autophagy. The overexpression and interference of ANXA2 were achieved through a hydrodynamic approach, which was previously proved effective for transmitting genes into kidneys [36, 42]. As the plasmids were delivered through the circulation, we could not exclude the non-renal effects of ANXA2, since ANXA2 was reported to play a role in immune and inflammation [43]. However, in cisplatin-induced AKI mice, we found ANXA2 increased primarily in renal tubules, although ANXA2 was also increased in the interstitial location of some immune cells, to a less extent. Therefore, we thought ANXA2 exerts protective effects mainly in renal tubular cells. Furthermore, we found ANXA2 promotes lysosomal function and autophagy in tubular cells in vivo and in vitro.

We further found ANXA2 induces TFEB transcription and expression. This depends on the activation of ANXA2 in β-catenin/TCF4 signaling. We did not test the phosphorylated status of TFEB although its subcellular localization could be modulated by its phosphorylation status [44], which is beyond the scope of this study. However, we observed ANXA2 induced the nuclear translocation of TFEB, representing the active form of TFEB [44]. We also found TFEB is a new downstream target of β-catenin. This was first recognized in our study, and this may explain the underlying mechanisms of β-catenin protecting against AKI [30].

As renal tubular epithelial cells have strong capabilities of repair and regeneration, hence, upon slight and mild insults, some tubular cells would undergo repair and regenerate through producing ANXA2. However, when the insults are strong and consistent, the upregulation of ANXA2 is not enough to resist the damage. Therefore, we thought ANXA2 represents a compensatory mechanism for tubular cell repair and regeneration. Indeed, we observed ANXA2 strongly protected against tubular cell apoptosis and AKI injury. This is related with its active role in β-catenin signaling. This is consistent with the previous observations, i.e., β-catenin and its downstream target MMP-7 are both increased in AKI and they inhibit the progression of AKI [30, 45, 46]. And this could also explain why ANXA2 inhibits AKI.

We adopted cisplatin-induced mouse models for its superiority in study on autophagy and lysosome functions in AKI [47, 48]. Cisplatin stimulation could induce a transient enhancement of autophagy and lysosomal function [47], however, it would damage autophagic activity, and decreased TFEB and lysosomal functions after a long-term exposure to kidneys [48, 49]. In this study, we found cisplatin inhibited lysosomal function and autophagy, although ANXA2 was concomitantly increased. This further indicates ANXA2 represents a compensatory mechanism for repair. Presumably, under a long-lasting stimulus, the increased ANXA2 would finally be not enough to compensate. We also collected the urinary samples from cisplatin-treated mice, and found that urinary ANXA2 was also increased in these animals. This suggests urinary ANXA2 would be potential to be an indicator of the biomarkers for AKI patients. Certainly, to investigate in a series of experimental models and a large cohort would be perfect to demonstrate it. This would be performed in the future.

Although more studies are needed, our result demonstrated proof of principle that ANXA2 could serve as a new potential for therapeutic strategies in cisplatin-induced AKI. Our studies also provided a new clue to better control AKI, i.e., to reasonably enhance lysosomal function would be a new check point to develop new therapeutic strategies for treating AKI.

## Materials and methods

### Cell line

Human proximal tubular cell line (HK-2) was purchased from the Cell Bank of the Chinese Academy of Sciences (Shanghai, China). Cells were maintained in DMEM/F12 culture medium supplemented with 10% fetal bovine serum and 1% penicillin/streptomycin (Gibco, CA, USA). Nuclear and cytoplasmic fractions were separated using a commercial kit (KeyGEN BioTECH, KPG1100, Jiangsu, China).

### Animal model

The animal experiments were approved by the Ethics Committee on Use and Care of Animals of Southern Medical University, Guangzhou, China. Male C57BL/6 mice (8-week-old) were purchased from the Experimental Animal Center of Southern Medical University (Guangzhou, China). Mice were kept in a standard 12-h light–dark cycle under the specific-pathogen-free condition and were allowed for free access to water and food. Mice were randomized into different groups using the online tool “Research Randomizer” (https://www.randomizer.org). 5 mice were included in each group to meet the minimum sample size requirement to perform an Analysis of Variance (ANOVA) test. The overexpression or interference of ANXA2 was achieved by a hydrodynamics-based gene delivery approach [36]. Briefly, the ANXA2 expression or shRNA plasmid or the empty vector was injected by tail vein at a dosage of 1.25 mg/kg. Cisplatin (Selleckchem, S1166) was administered by a single intraperitoneal injection (25 mg/kg). Mice were sacrificed 48 h after cisplatin injection. Kidney tissues and serum samples were collected for the following assessment.

### Serum creatinine and urea nitrogen assessment

The concentrations of serum creatinine and urea nitrogen were assessed using an automatic chemistry analyzer (AU480, Chemistry Analyzer, Beckman Coulter, Atlanta, USA).

### Plasmid and lentivirus construction

All constructs were constructed and verified by DNA sequencing (Genecreate Biological Engineering, Wuhan, China). Primers used for plasmid construction are available upon request. The RFP-GFP-LC3B lentivirus was purchased from HanBio Technology (HANBIO, HB-LP2100001, Shanghai, China).

### Terminal deoxynucleotidyl transferase dUTP Nick-End labeling (TUNEL) assay

The TUNEL assay was carried out using the TUNEL apoptosis Assay Kit (Beyotime Institute of Biotechnology, C1086, China) according to the manufacturer’s protocol, and counter-stained with Hoechst 33258 (Beyotime Institute of Biotechnology, China). Images were taken by fluorescent microscopy (Leica TCS SP2 AOBS, Leica Microsystems, Cambridge, UK).

### Flow cytometry analysis of apoptosis

HK-2 cells were stained with the PE/Annexin V Apoptosis Detection Kit (BD, 559763, USA) and analyzed using BD LSR fortessa X-20 (USA) flow cytometry.

### Western blotting and immunoprecipitation

Protein concentrations were measured using BCA kit (BioVision K813-5000 Milpitas, CA). Protein samples (15-40 μg) were subjected to SDS-PAGE electrophoresis and then were electro-transferred to a 0.45 μm polyvinylidene difluoride membrane (Merck Millipore Ltd, IPVH00010, Ireland). The membrane was blocked and incubated with primary antibodies, secondary horseradish peroxidase-conjugated antibody, and visualized with an ECL kit (Applygen, p1020, Beijing, China). For Co-IP assay, 200 μg of cellular protein was incubated with primary antibody, and precipitated using agarose beads (Santa Cruz biotechnology, sc-2003, USA). Antibodies were shown in Supplementary Table S1.

### Quantitative real-time-polymerase chain reaction (qrtPCR)

Total RNA was isolated using Trizol (Invitrogen, 15596026), and reacted in a 20 μL system using HiScript III RT superMix (+gDNA wiper) (vazyme^TM^, R323-01). Two microliters of cDNA were amplified by qrtPCR with specific primers (Supplementary Table S2) using SYBR qPCR Master Mix (High ROX Premixed) (vazyme^TM^, Q341-02/03) in an ABI PRISM 7000 Sequence Detection System (Applied Biosystems, Foster City, CA).

### Chromatin immunoprecipitation (ChIP)

ChIP assays were performed using SimpleChIP Plus Enzymatic Chromatin IP Kit (Magnetic Beads) (Cell Signaling TECHNOLOGY, 38191, USA). Relative antibodies and sequence of primers used were described in the supplementary documents (Supplementary Table S2).

### Luciferase analysis

HK-2 cells were co-transfected with renilla luciferase, pGL3-TFEB promoter reporter plasmid, and ANXA2 or TCF4 expression plasmid using Lipofectamine 2000 (11668-019, Invitrogen, USA). Cells were assayed using the Dual-Luciferase Reporter Assay System (Dual-luciferase Report Assay System, E1910, Promega, USA).

### Pathological and immunohistochemical staining

Paraffin-embedded kidney sections were stained by periodic acid-Schiff staining according to standard procedure. Immunohistochemical and immunofluorescence staining were performed in paraffin and frozen sections. The slides were incubated with primary antibodies and the secondary antibodies and visualized using the ABC (VECTA ABC Kit, Peroxidase, PK-6100, USA), AEC (VECTASTAIN Elite ABC Kit, Peroxidase PK-6100, USA) or DAB kit (Vector Laboratories SK-4100, USA). For immunofluorescence staining, the fluorophore-conjugated secondary antibody was added to the slides, which were counter-stained with DAPI to observe by confocal microscope (Leica Microsystems CMS GmbH, TCS SP8 X, Germany).

### Transmission electron microscopy (TEM)

Kidney tissues or HK-2 cells were collected and fixed in 1.25% glutaraldehyde (0.1 mol/L) in phosphate buffer. Ultrathin sections (60 nm) were prepared by a routine procedure and were examined under an electron microscope (JEOL JEM-1010, Tokyo, Japan).

### Detection of autophagic flux

HK-2 cells were transfected with RFP-GFP-LC3B lentivirus (HANBIO, HB-LP2100001, Shanghai, China). Autophagosomes and autophagolysosomes were observed using confocal microscope (Leica Microsystems CMS GmbH, TCS SP8 X, Germany). The RFP-GFP-LC3B sensor enables detection of neutral-pH LC3B-positive autophagosomes (green fluorescence) and acid-pH LC3B-positive autophagolysosomes (red fluorescence).

### Detection of lysosome

Live cells were incubated with Lysosome Tracker Red (50 nmol/L, Beyotime, China, C1046) for 10 min and counterstained with Hoechst 33432 (1 μg/mL, Beyotime, China, C1042).

### RNA sequencing and bioinformatics analysis

RNA sequencing was performed by Applied Protein Technology (Shanghai, China). Briefly, qualified RNA samples were used for paired-end library construction using the ABclonal mRNA-seq Lib Prep Kit (ABclonal, China). PCR products were then purified (AMPure XP system). Genome sequencing was performed with an Illumina Novaseq 6000/MGISEQ-T7 instrument.

### Quantifications of staining

Immunohistochemical, immunofluorescence were quantified at high-powered (×400, ×1000) fields from randomly selected 5 fields each section. Quantification of positive staining was assessed by two researchers who were blinded through Image Pro Plus software.

### Statistical analysis

All of the data are expressed as the mean ± standard error of the mean (SEM). Statistical analysis was carried out using SPSS 20.0 (SPSS Inc, Chicago, IL). The Student’s *t* test or *t*′, test was used to compare the means between two groups. ANOVA was used to compare the means among three or more groups, followed by the Least Square Difference *post hoc* test when the variance between groups was homogeneous, or the Dunnett T3 test when the variance between groups was not homogeneous. A *P* value < 0.05 was considered to be statistically significant.

## Supplementary information

Supplementary Data

Author Contribution Statement

Original Data File

## Data Availability

Sequencing data produced in this study have been uploaded to the NCBI SRA database (accession number: PRJNA806160). Full unedited gels and blots produced in this study have been provided in the Supplementary material. Other raw data supporting the conclusion of this article will be made available by the authors, without undue reservation, to any qualified researcher.
