# Supplementary material for: Annexin A2 plays a key role in protecting against cisplatin-induced AKI through β-catenin/TFEB pathway
Source: Cell Death Discov. 2022 Oct 28;8:430. doi: 10.1038/s41420-022-01224-w (PMC9616836; doi:10.1038/s41420-022-01224-w)
Supplement: Supplementary file 1 — Supplementary Data [file 41420_2022_1224_MOESM1_ESM.docx]

**Supplementary Figure S1**

The regulatory effects ANXA2 on autophagy, lysosomal functions and Wnt/β-catenin signaling


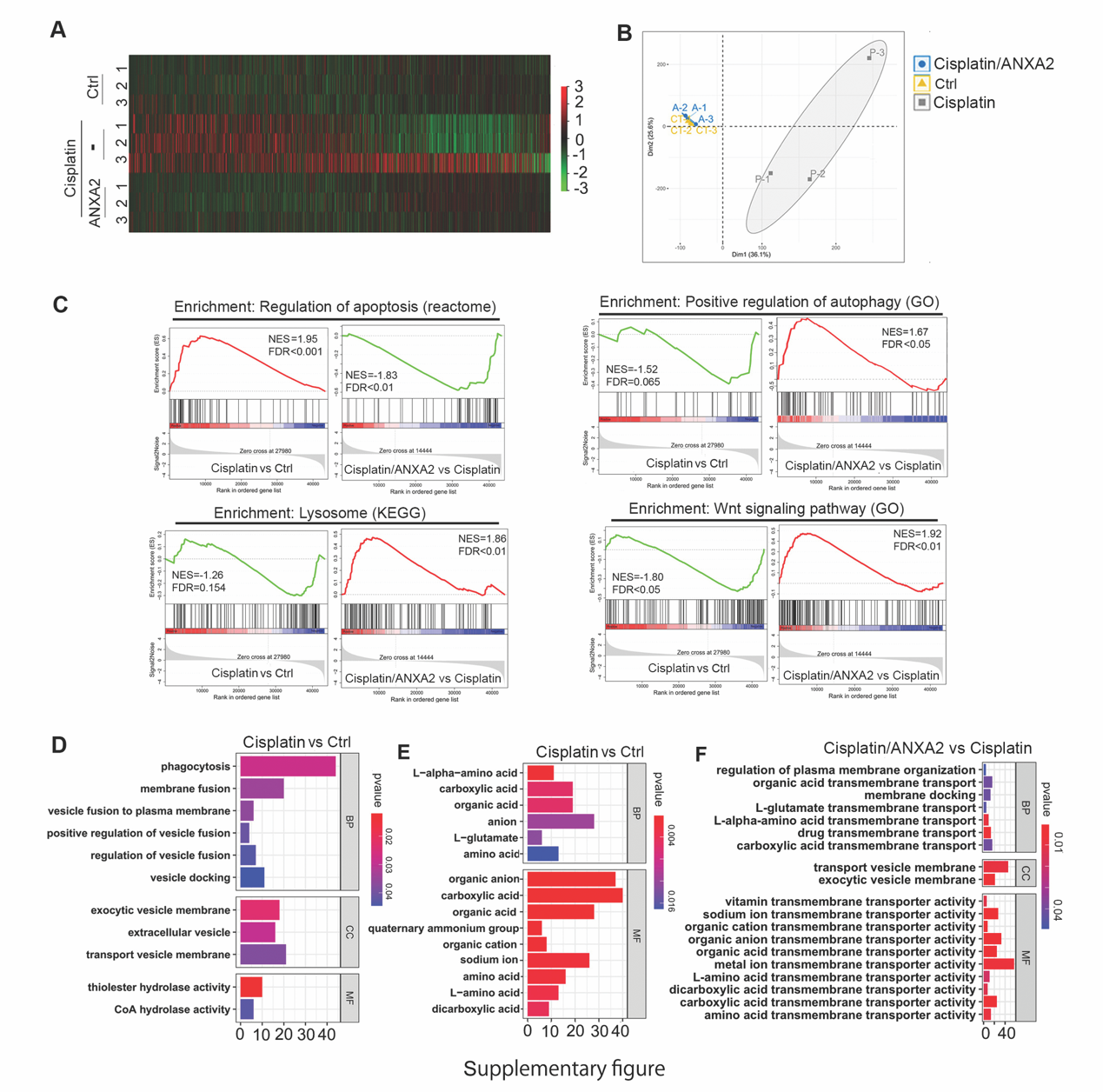


(A) Significant DEGs (|log_2_FC|≥1, adjusted p value<0.05) between the groups “pcDNA3” and “ANXA2” are visualized in the heatmap with corresponding gene expression in the group “Ctrl” being listed by the side. The color ranges from red to green represents the log2 ranked FPKM value from large to small. (B) The PCA biplot shows the degree of the intragroup replication and the intergroup difference. Distance between points indicates sample difference. (C) GSEA demonstrates enrichment of gene sets related to apoptosis, positive regulation of autophagy, lysosome and Wnt signaling pathway. Left panels: positive NES indicates higher expression in the group “pcDNA3” versus the group “Ctrl”; negative NES indicates lower expression in the group “pcDNA3” versus the group “Ctrl”. Right panels: positive NES indicates higher expression in the group “ANXA2” versus the group “pcDNA3”; negative NES indicates lower expression in the group “ANXA2” versus the group “pcDNA3”. (D) Enrichment analysis was performed on significant DEGs between the groups “pcDNA3” and “Ctrl”. Enriched GO terms related to the lysosomal biological processes, structure and functions are visualized in the bar graph. The number appeared on the x-axis indicates the number of enriched genes in a GO category. The color ranges from red to blue represents the significance of enrichment from more to less. (E) Enrichment analysis was performed on significant DEGs between the groups “pcDNA3” and “Ctrl”. Enriched GO terms related to the “transmembrane transport-BP” or “transmembrane transporter activity-MF” are visualized in the bar graph with the transported chemical names being listed in the left. The number appeared on the x-axis indicates the number of enriched genes in a GO category. The color ranges from red to blue represents the significance of enrichment from more to less. (F) Enrichment analysis was performed on significant DEGs between the groups “ANXA2” and “pcDNA3”. Enriched GO terms are visualized in the bar graph. The number appeared on the x-axis indicates the number of enriched genes in a GO category. The color ranges from red to blue represents the significance of enrichment from more to less. Abbreviations: ANXA2, ANXA2; BP, biological process; CC, cellular component; Ctrl, control; FDR, false discovery fate; FPKM, fragments per kilobase of transcript; GO, gene ontology; GSEA, gene set Enrichment Analysis; KEGG, Kyoto Encyclopedia of Genes and Genomes; MF, molecular function, NES, normalized enrichment score; PCA, principle component analysis; pcDNA3, empty vector pcDNA.3; Wnt, wingless/integrated.

**Supplementary Table S1**

Antibody list

| Antibodies used for western blotting (WB), immunoprecipitation (IP) and immunofluorescence (IF) | | | | |
| --- | --- | --- | --- | --- |
| Protein | Applications | Antibody | Origin | Dilution |
| TCF4 | IP | 2565, CST | Rabbit | 1:1000, 1:50 |
| Annexin A2 | WB, IF | Ab178677, Abcam | Rabbit | 1:50,000, 1:40, 1:50 |
| β-catenin | WB, IHC | 610154,BD | Mouse | 1:10,000, 1:100 |
| active-β-catenin | WB | 19807T, CST | Rabbit | 1:1000 |
| P-mTOR | WB | Ab109268, Abcam | Rabbit | 1:1000 |
| P62 | WB | 5114, CST | Rabbit | 1:1000 |
| Kim-1 | IHC | AF1817, R&D | Goat | 1:50 |
| TFEB | WB, IF, IHC | 13372-1-AP, Proteintech | Rabbit | 1:1000, 1:50, 1:50 |
| LAMP1 | WB, IF | Ab208943, abcam | Rabbit | 1:1000, 1:100 |
| LAMP2 | WB | Ab203224, abcam | Rabbit | 1:1000 |
| ATG5 | WB | Ab108327, Abcam | Rabbit | 1:1000 |
| PARP | WB | 9542S, CST | Rabbit | 1:1000 |
| LC3B | WB | Ab128025, Abcam | Rabbit | 1:1000 |
| LC3B | IF | 43566, CST | Rabbit | 1:1000 |
| Cleaved caspase-3 | WB | 9661S, CST | Rabbit | 1:1000 |
| Fasl | WB | sc19681, Santa | Mouse | 1:500 |
| Bax | WB | sc7480, Santa | Mouse | 1:500 |
| Bcl2 | WB | 15071, CST | Rabbit | 1:1000 |
| α-Tubulin | WB | RM2007,Ray antibody | Mouse | 1:5000 |
| GAPDH | WB | RM2002,  Ray antibody | Mouse | 1:5000 |
| β-actin | WB | RM2001,  Ray antibody | Mouse | 1:5000 |
| Normal rabbit IgG | IP | 2729, CST | Rabbit | 1: 500 |
| Histone H3 | IP | 4620, CST | Rabbit | 1: 50 |
| TBP | WB | Ab818，Abcam | Mouse | 1:1000 |

**Supplementary Table S2**

Primer sequences

| Gene names | Sequences |
| --- | --- |
| Sequences of primers used for Quantitative real-time-PCR | |
| Ms-Anxa1-F | AAGGTGTGGATGAAGCAACCAT |
| Ms-Anxa1-R | TGCATCAAACTGAGCTGGAGTT |
| Ms-Anxa2-F | ACATTGCCTTCGCCTATCAGA |
| Ms-Anxa2-R | CATCATACTGGGCAGGTGTCT |
| Ms-Anxa3-F | TCAAGCAGGCAGATGAAGGA |
| Ms-Anxa3-R | TGGCCAGATGTTCATCCACT |
| Ms-Anxa4-F | TTGAGGACTTGAAGTCGGAGC |
| Ms-Anxa4-R | CGAGAGGCCAGGATCTCAATC |
| Ms-Anxa5-F | GCTGATGCAGAAGTCCTTCG |
| Ms-Anxa5-R | TCAGGTCATCCACAAGGTCC |
| Ms-Anxa6-F | TGGCAAGGCTGATTCTGGG |
| Ms-Anxa6-R | TGGCCCGGATTTCAGCATT |
| Ms-Anxa7-F | AGCCACCTGCACAGTCTTAT |
| Ms-Anxa7-R | TAGGGTAGGGAGCTTGTCCT |
| Ms-Anxa8-F | GCAGGGACACGTGATGGAA |
| Ms-Anxa8-R | TGCTGCTGAGAGTCTTGCC |
| Ms-Anxa9-F | AAGCATGACTTCCAGGTGGAG |
| Ms-Anxa9-R | CCCGGACATCTTGTTCTTCCA |
| Ms-Anxa10-F | CCTAACACAACGCAGCAATGC |
| Ms-Anxa10-R | CATCAGGCCCACCATCACTTC |
| Ms-Anxa11-F | ATGAGGGGAGCAGGAACAAAG |
| Ms-Anxa11-R | GGTACAGTGACTTGCCGTACA |
| Ms-Anxa13-F | ATTGTGACCAGAGCAGAGGTG |
| Ms-Anxa13-R | TACCAGCAGTTTCCGGAAGTC |
| Ms-atp6ap1-F | GGCAACAGTGGTATCTCGGAT |
| Ms-atp6ap1-R | GCCACCAACGACAGAGACAG |
| Ms-Lamp1-F | CAGCACTCTTTGAGGTGAAAAAC |
| Ms-Lamp1-R | CCATTCGCAGTCTCGTAGGTG |
| Ms-Lamp2-F | GCACAGTGAGCACAAATGAGT |
| Ms-Lamp2-R | CAGTGGTGTGTATGGTGGGT |
| Ms-PSAP-F | TGCTGAAAGATAATGCTACGCA |
| Ms-PSAP-R | GCAGGTAAGAGTCAACCACCTC |
| Ms-TFEB-F | GCTCCAACCCCGAGAAAGAG |
| Ms-TFEB-R | CAGCGTGTTAGGCATCTGC |
| Ms-TPP1-F | GGCAAATGCACTTACAACCCT |
| Ms-TPP1-R | CTCCGAGAGTCTTTCCAGGT |
| Ms-β actin-F | GAGCGCAAGTACTCTGTGTG |
| Ms-β actin-R | AACGCAGCTCAGTAACAGTC |
| Hu-TFEB-F | ACCTGTCCGAGACCTATGGG |
| Hu-TFEB-R | CGTCCAGACGCATAATGTTGTC |
| Hu-ANXA2-F | ACATTGCCTTCGCCTATCAGA |
| Hu-ANXA2-R | CATCATACTGGGCAGGTGTCT |
| Hu-Lamp1-F | CAGATGTGTTAGTGGCACCCA |
| Hu-Lamp2-R | TTGGAAAGGTACGCCTGGATG |
| Hu-PASP-F | CCCGGTCCTTGGACTGAAAG |
| Hu-PASP-R | TATGTCGCAGGGAAGGGATTT |
| Hu-Atp6ap1-F | CAGCGACTTGCAGCTCTCTAC |
| Hu-Atp6ap1-R | TGAAATCCTCAATGCTCAGCTTG |
| Hu-CTSA-F | GTCGCCCAGAGCAATTTTGAG |
| Hu-CTSA-R | TCTCCCCGGTCAGGAAAAGTT |
| Hu-TPP1-F | CCTCCACACGGTGCAAAAATG |
| Hu-TPP1-R | CTCTGCTTGTCGGATGCTCAG |
| Hu-β actin-F | GCATCAAACTGAGCTGGAGTT |
| Hu-β actin-R | CATCATACTGGGCAGGTGTCT |
| Sequences of siRNA used for cell transfection | |
| Hu-TCF4siRNA-1-F | GCACUUGCUUCGAUCUAUUTT |
| Hu-TCF4siRNA-1-R | AAUAGAUCGAAGCAAGUGCTT |
| Hu-TCF4siRNA-2-F | GCAACCAGCACUUUCCCUATT |
| Hu-TCF4siRNA-2-R | UAGGGAAAGUGCUGGUUGCTT |
| Hu-TCF4siRNA-3-F | GGUACAGACAAAGAAAGUUTT |
| Hu-TCF4siRNA-3-R | AACUUUCUUUGUCUGUACCTT |
| Hu-ANXA2siRNA-F | GACCAACCGCAGCAAUGCATT |
| Hu-ANXA2siRNA-R | UGCAUUGCUGCGGUUGGUCTT |
| Negative control 5’to 3’  (sense) | UUCUCCGAACGUGUCACGUTT |
| Negative control 5’to 3’  (antisense) | ACGUGACACGUUCGGAGAATT |
| Sequences of primers used for plasmid vector | |
| pLVX-shRNA2-LKO.1 5’ | GACTATCATATGCTGTACCGT |
| PCDNA3.1(+)-F | CTAGAGAACCCACTGCTTAC |
| PCDNA3.1(+)-R(BGH-R) | TAGAAGGCACAGTCGAGG |
| Sequences of primers used for ChIP-PCR | |
| SimpleChiP human RPL30 Exon 3 primers | Cell Signaling TECHNOLOGY, 7014 (confidential sequence) |
| Hu-TFEB promoter-F | ATCATGGGGAGAGGAGACCC |
| Hu-TFEB promoter-R | AATGCCCTAGGAGGGTGCAG |
