# Supplementary material for: Annexin A2 plays a key role in protecting against cisplatin-induced AKI through β-catenin/TFEB pathway
Source: Cell Death Discov. 2022 Oct 28;8:430. doi: 10.1038/s41420-022-01224-w (PMC9616836; doi:10.1038/s41420-022-01224-w)
Supplement: Supplementary file 2 — Author Contribution Statement [file 41420_2022_1224_MOESM2_ESM.docx]

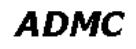


**DECLARATION** **OF** **CONTRIBUTIONS** **TO** **ARTICLE**

Kunyu Shen, Jinhua Miao, Qiongdan Gao, Xian Ling, Ye Liang, Qin Zhou, Qirong Song, Yuxin Luo, Qinyu Wu, Weiwei Shen, Xiaonan Wang, Xiaolong Li, Youhua Liu, Shan Zhou, Ying Tang, Lili Zhou

*Cell Death Discovery*

Annexin A2 plays a key role in protecting against cisplatin-induced AKI through β-catenin/TFEB pathway


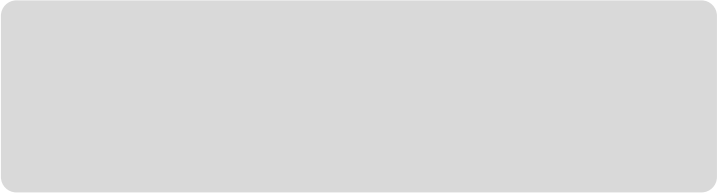


| \| Manuscript Number: \| \| \| --- \| --- \| \|  \| **CDDISCOVERY-22-4699-R** \| \| Proposed Title of the Contribution:  Author(s): \| \|   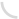 | (the ‘Journal’)  (the ‘Contribution)  (the ‘Authors’) |
| --- | --- | --- | --- | --- | --- | --- | --- |

Journal Name:

For all *CDD* articles, each person named as an author in the published version must be able to show he or she has contributed substantially to the article.

Authorship credit should be based on 1) substantial contributions to conception and design, acquisition of data, or analysis and interpretation of data; 2) drafting the article or revising it critically for important intellectual content; and 3) final approval of the version to be published. Authors should meet conditions 1, 2 and 3.

Any person who cannot be shown to have made a substantial contribution to the article cannot be listed as an author in the final version. The name of any person who is deemed to have made a minor contribution can, however, appear in the Acknowledgments section of the article.

Please complete the table below to indicate the contributions of all named authors to the manuscript.

Specification of Contribution to the Manuscript:

Aquisition of data, data analysis, drafting the manuscript, final approval of the version to be published

| Kunyu Shen |
| --- |

Aquisition of data, data analysis, revising the manuscript, final approval of the version to be published

| Jinhua Miao |
| --- |

Aquisition of data, revising the manuscript, final approval of the version to be published

| Qiongdan Gao |
| --- |

Aquisition of data, revising the manuscript, final approval of the version to be published

Xian Ling

|  |
| --- |

Aquisition of data, revising the manuscript, final approval of the version to be published

Ye Liang

|  |
| --- |

Aquisition of data, revising the manuscript, final approval of the version to be published

| Qin Zhou |
| --- |

Qirong Song

| Aquisition of data, revising the manuscript, final approval of the version to be published |
| --- |

Aquisition of data, revising the manuscript, final approval of the version to be published

| Yuxin Luo |
| --- |

Interpretation of data, revising the manuscript, final approval of the version to be published

| Qinyu Wu |
| --- |

Aquisition of data, revising the manuscript, final approval of the version to be published

Weiwei Shen

|  |
| --- |

| Interpretation of data, revising the manuscript, final approval of the version to be published  Xiaonan Wang |
| --- |

Interpretation of data, revising the manuscript, final approval of the version to be published

Xiaolong Li

|  |
| --- |

| Interpretation of data, revising the manuscript, final approval of the version to be published  Youhua Liu |
| --- |

conception and design, data analysis, revising the manuscript, approval of the final version

Shan Zhou

conception and design, data analysis, revising the manuscript, approval of the final version

Ying Tang

conception and design, data analysis, revising the manuscript, approval of the final version

Lili Zhou

Please complete the table below to indicate the contributions of all named authors to the figures

Figure

1:

| Aquisition of data: Kunyu Shen, Jinhua Miao  Statistics: Xian Ling, Ye Liang  Interpretation of result: Lili Zhou |
| --- |

Figure 2:

Aquisition of data: Qirong Song, Yuxin Luo

Statistics: Qin Zhou

Interpretation of result: Ying Tang

|  |
| --- |

Figure 3:

| Aquisition of data: Kunyu Shen, Jinhua Miao, Weiwei Shen  Statistics: Xian Ling, Ye Liang  Interpretation of result: Shan Zhou |
| --- |

Figure 4:

Aquisition of data: Kunyu Shen, Jinhua Miao, Qinyu Wu

Statistics: Xian Ling, Ye Liang

Interpretation of result: Shan Zhou

|  |
| --- |

Figure 5:

| Aquisition of data: Kunyu Shen, Jinhua Miao, Xiaonan Wang  Statistics: Xian Ling, Ye Liang  Interpretation of result: Lili Zhou |
| --- |

Figure 6:

Aquisition of data: Kunyu Shen, Jinhua Miao, Xiaolong Li

Statistics: Xian Ling, Ye Liang

Interpretation of result: Lili Zhou

|  |
| --- |

Figure 7:

Aquisition of data: Kunyu Shen, Jinhua Miao

Statistics: Xian Ling, Ye Liang, Shan Zhou

Interpretation of result: Lili Zhou

Figure 8:

Aquisition of data: Kunyu Shen, Jinhua Miao, Qiongdan Gao

Statistics: Xian Ling, Ye Liang, Kunyu Shen

Interpretation of result: Lili Zhou, Shan Zhou

Figure 9:

Aquisition of data: Kunyu Shen, Jinhua Miao, Qiongdan Gao

Statistics: Xian Ling, Ye Liang, Kunyu Shen

Interpretation of result: Lili Zhou, Shan Zhou, Youhua Liu, Kunyu Shen

|  | Signed for and on behalf of the Author(s):   \| 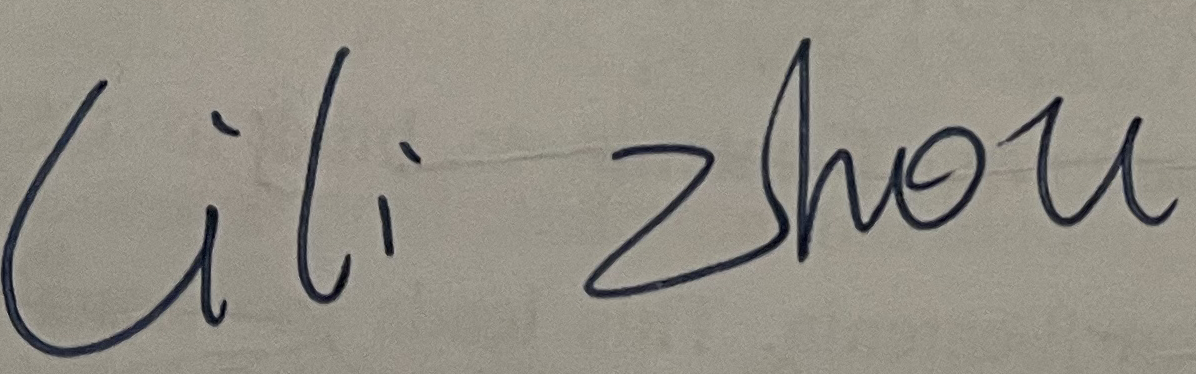 \| \| --- \| | Print Name:   \| LILI ZHOU \| \| --- \| | Date*:*   \| 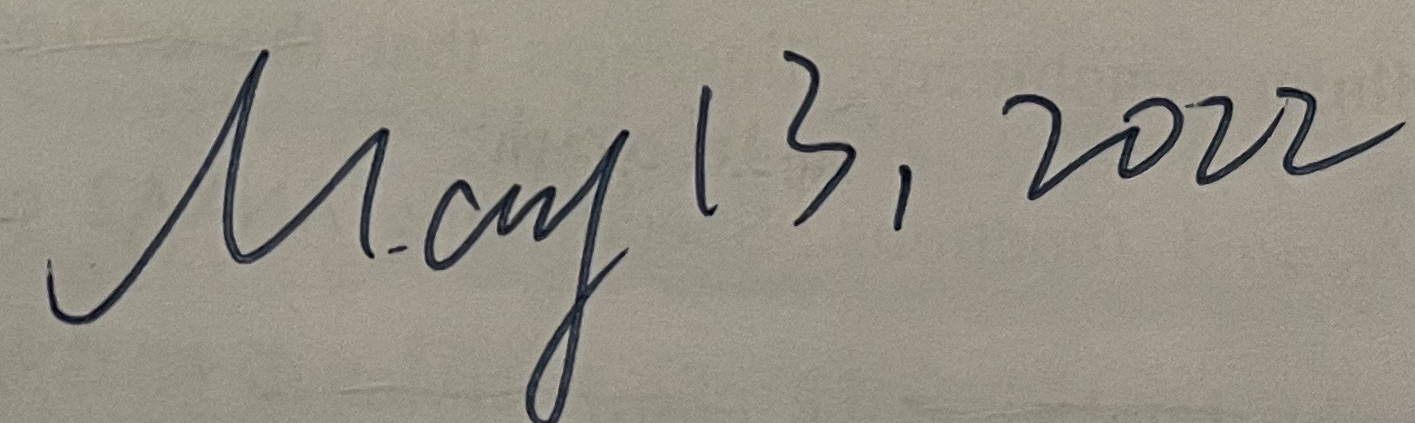 \| \| --- \| |  |
| --- | --- | --- | --- | --- | --- | --- | --- |
