# Supplementary material for: Annexin A2 plays a key role in protecting against cisplatin-induced AKI through β-catenin/TFEB pathway
Source: Cell Death Discov. 2022 Oct 28;8:430. doi: 10.1038/s41420-022-01224-w (PMC9616836; doi:10.1038/s41420-022-01224-w)

Full unedited gels for Figure 1

1B

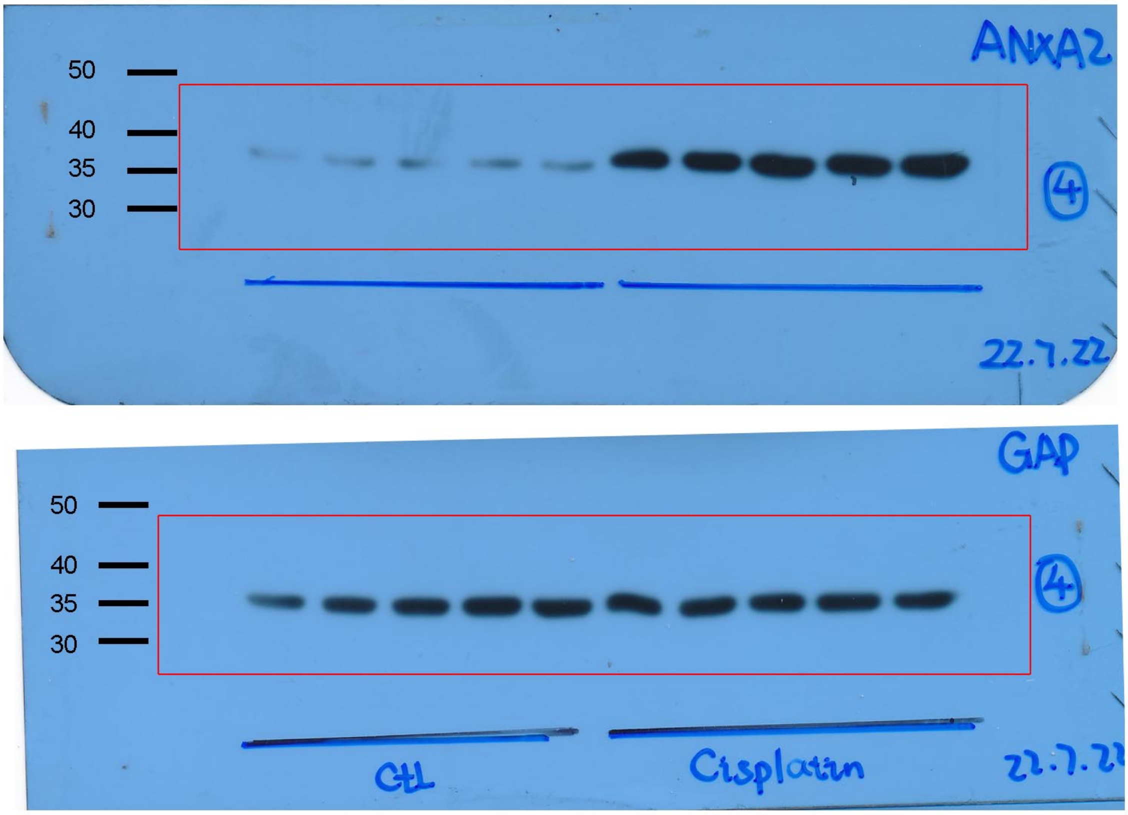

1I

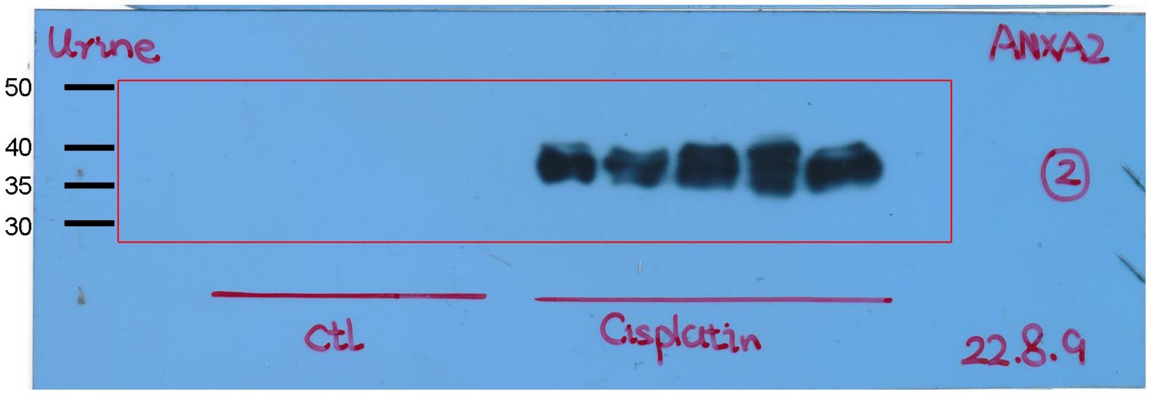

Full unedited gels for Figure 2F

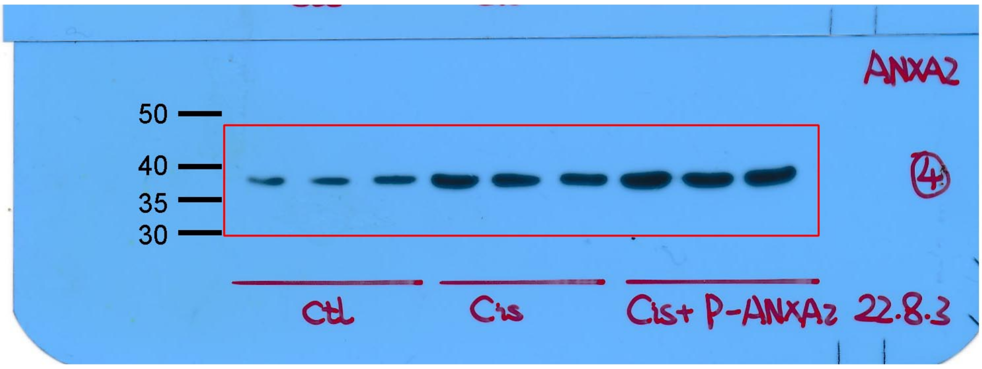

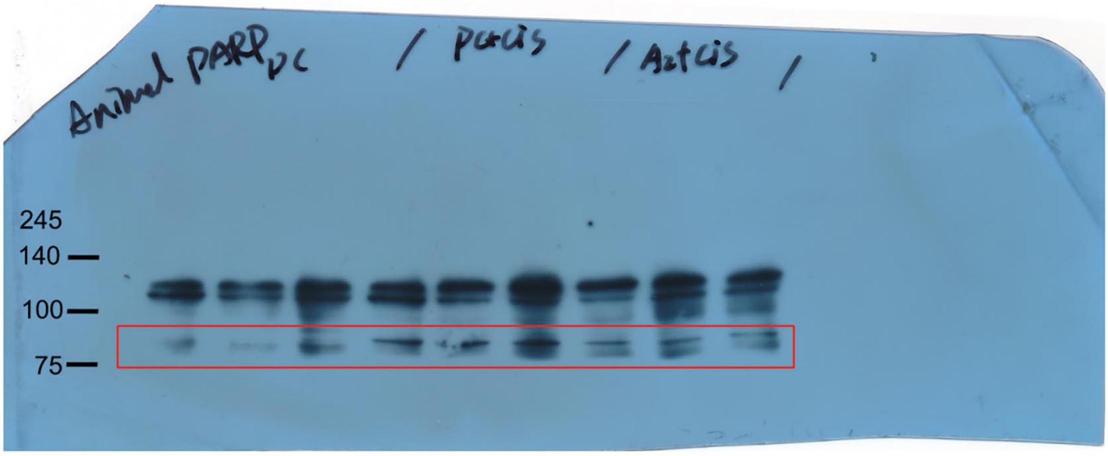

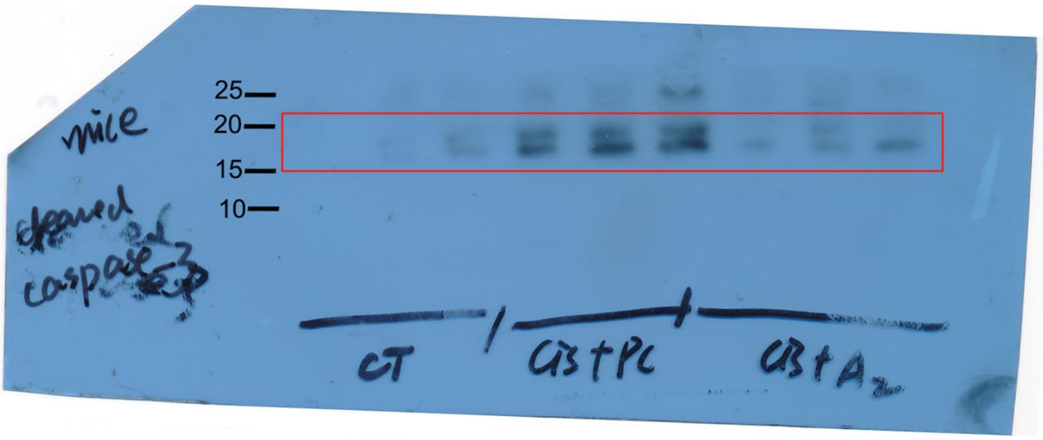

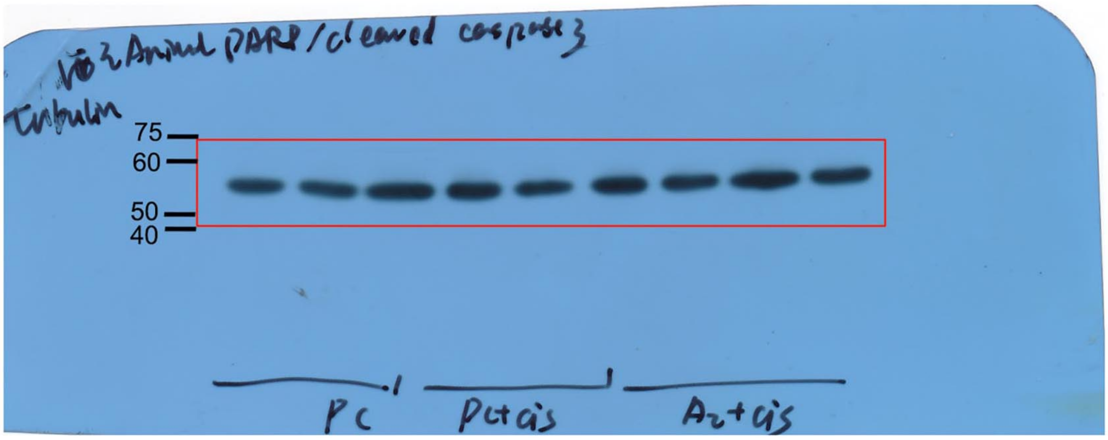

Full unedited gels for Figure 3D

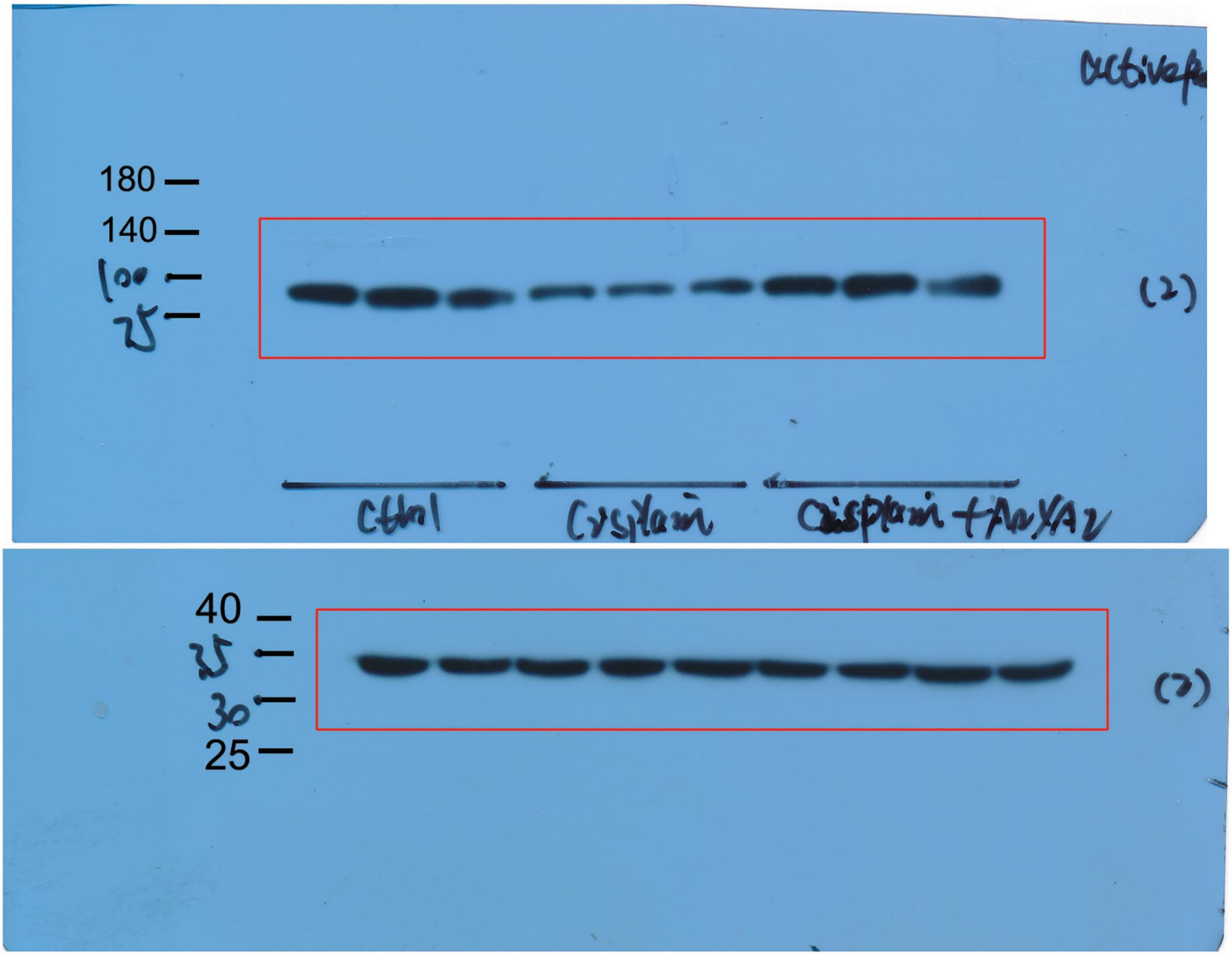

Full unedited gels for Figure 3F

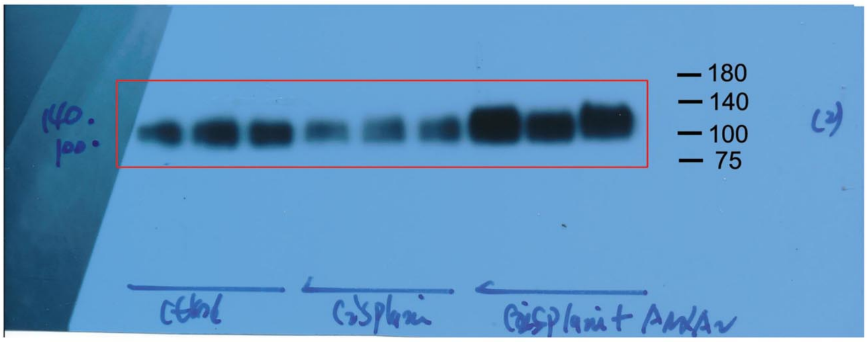

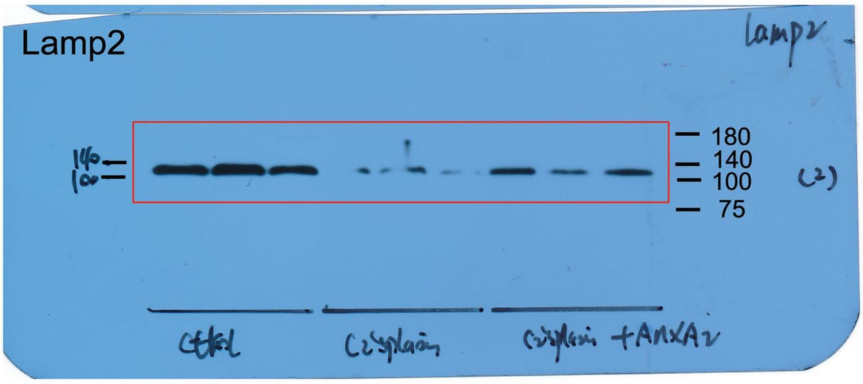

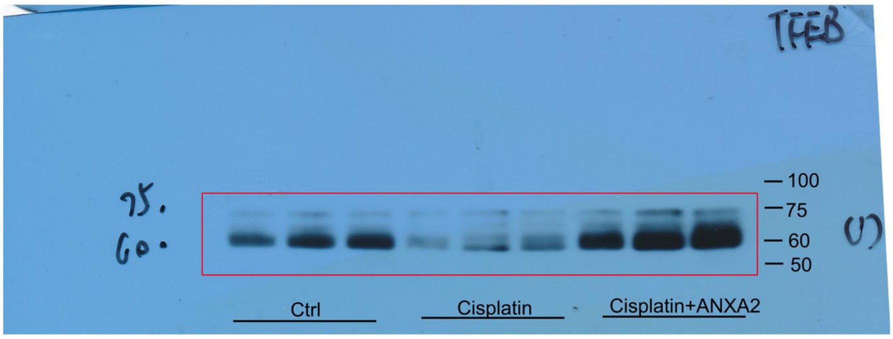

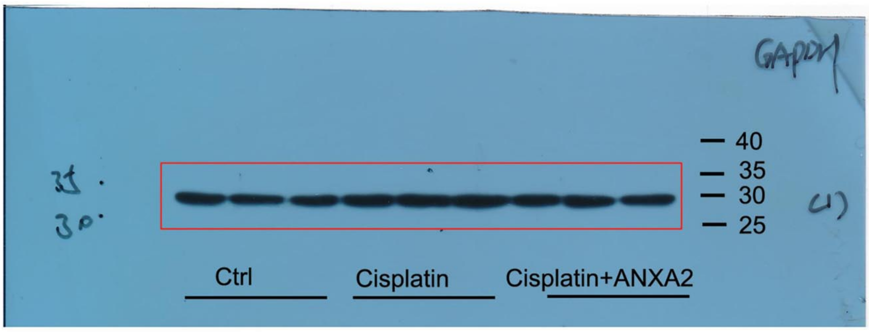

Full unedited gels for Figure 4C

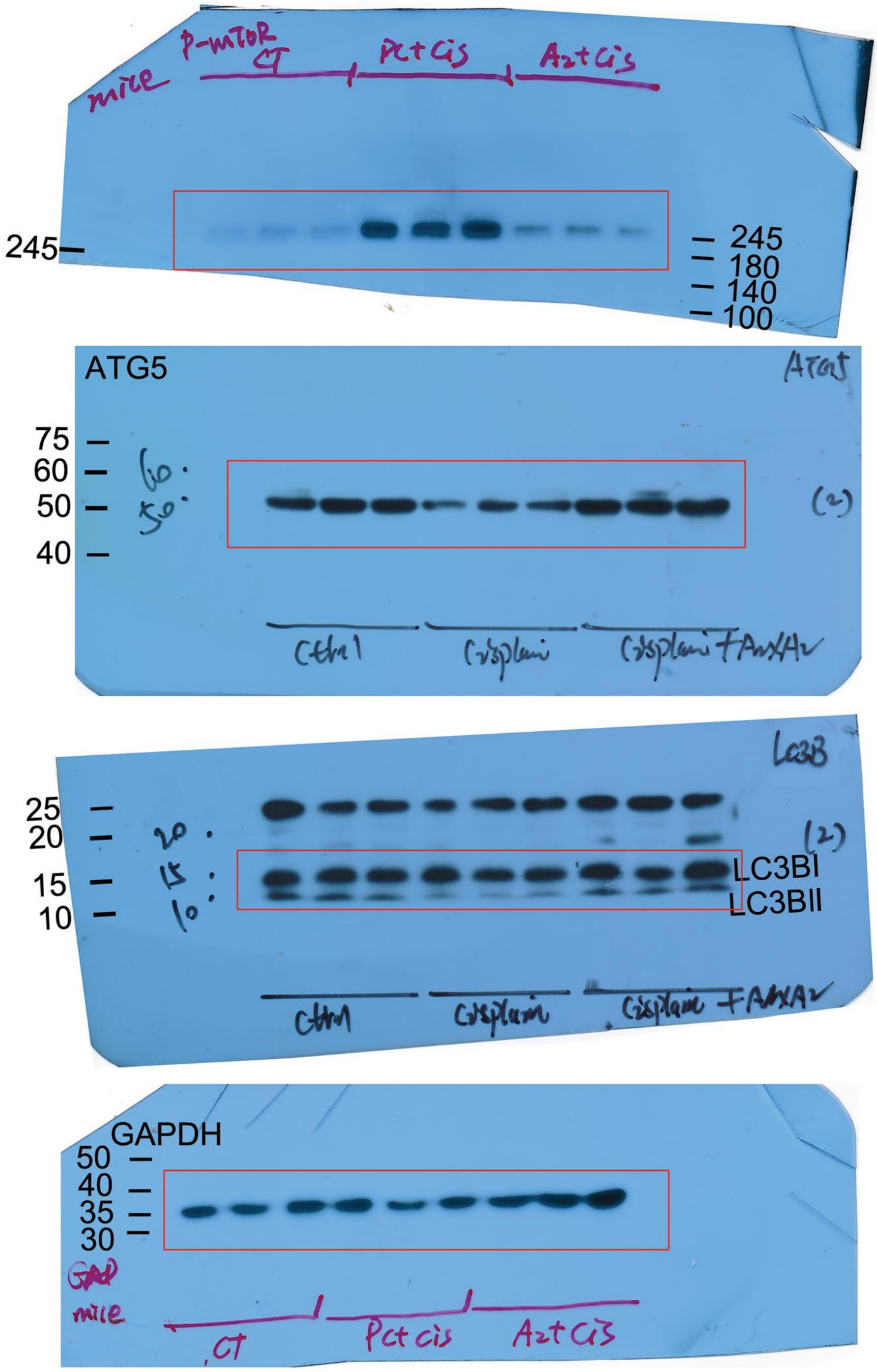

Full unedited gels for Figure 5H

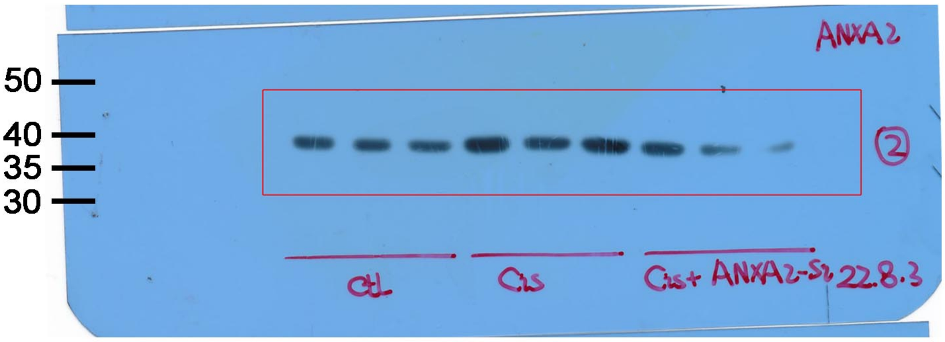

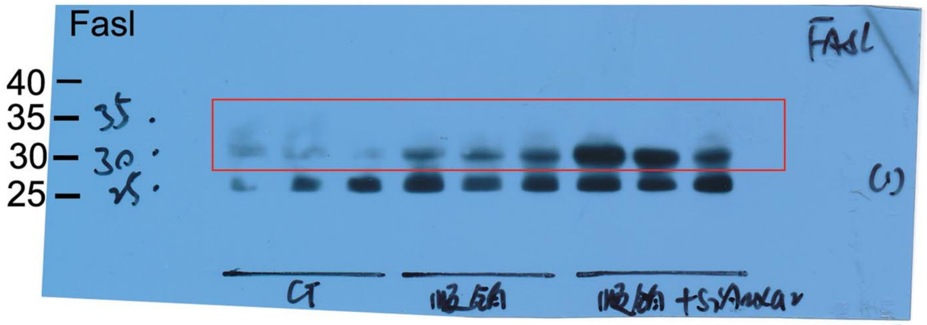

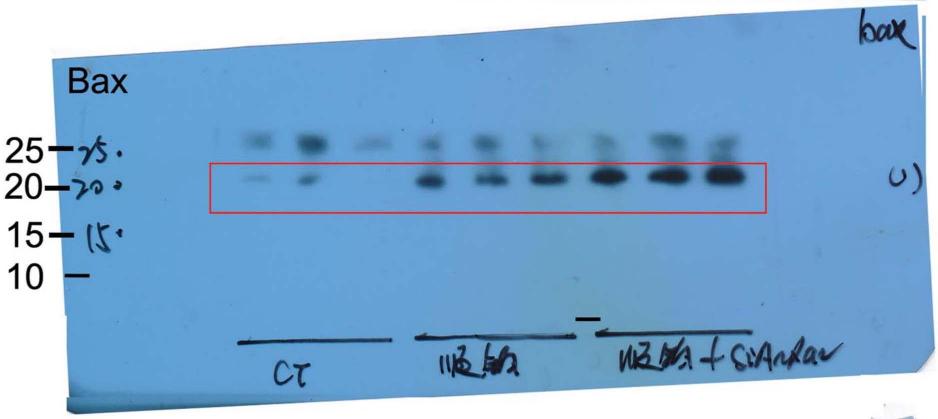

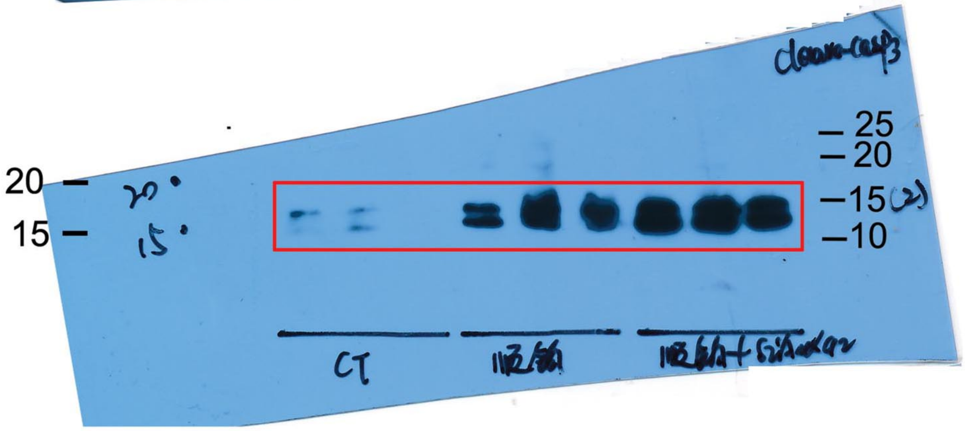

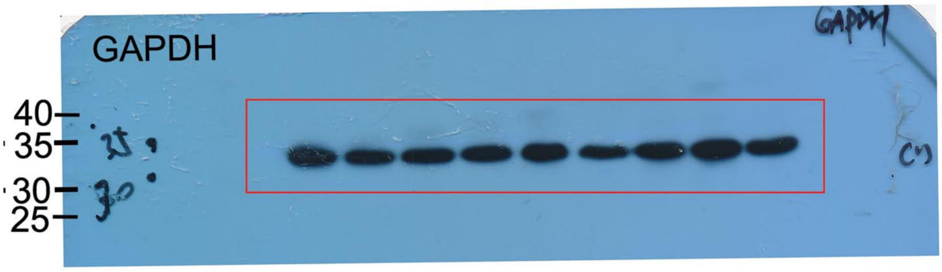

Full unedited gels for Figure 6A

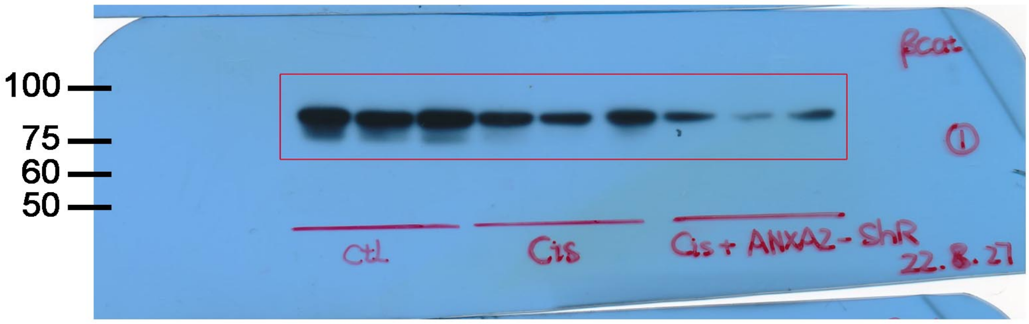

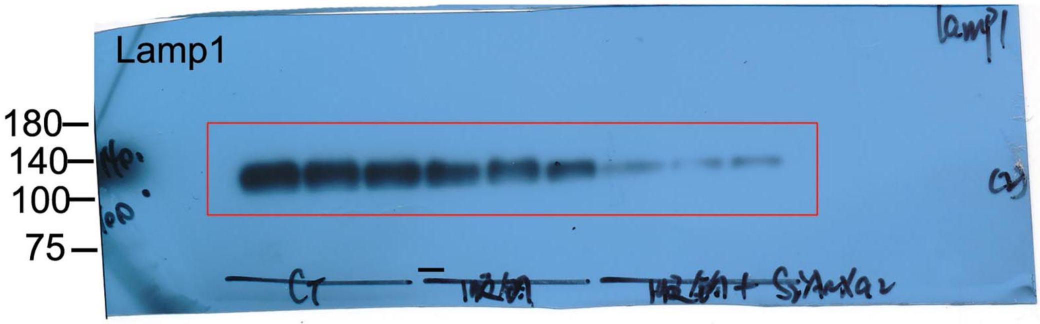

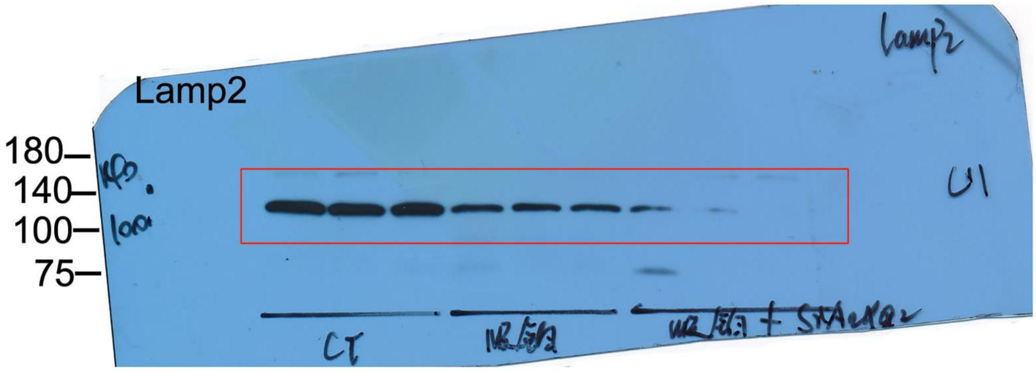

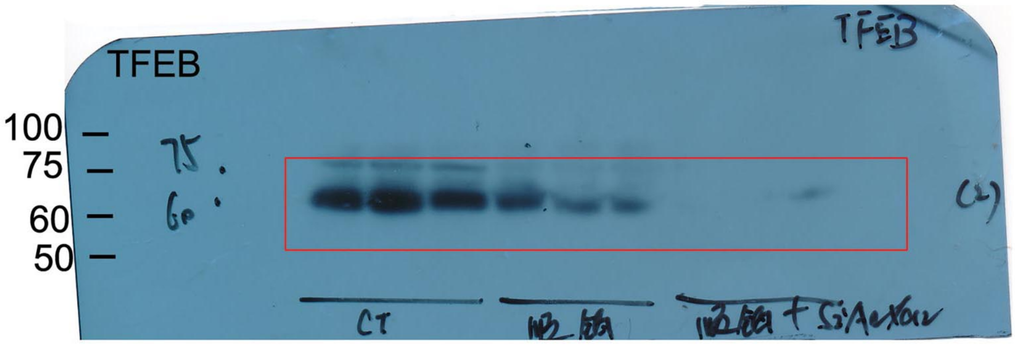

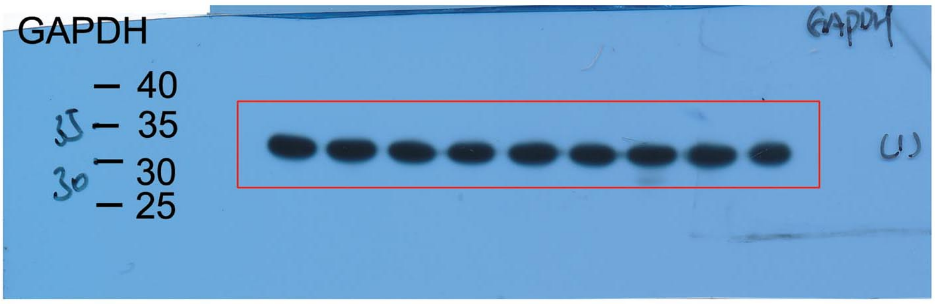

Full unedited gels for Figure 6N

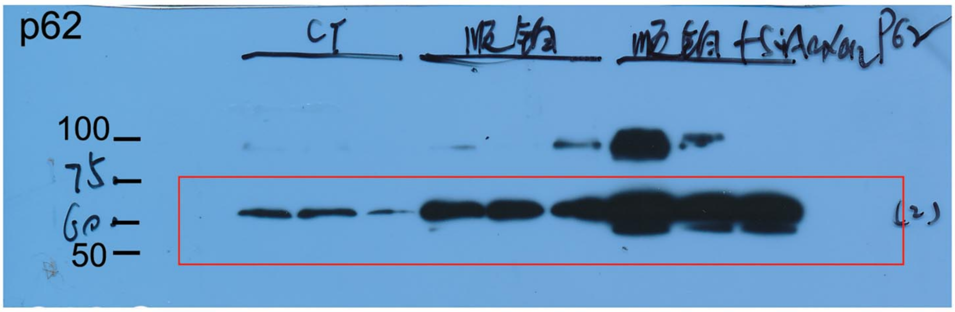

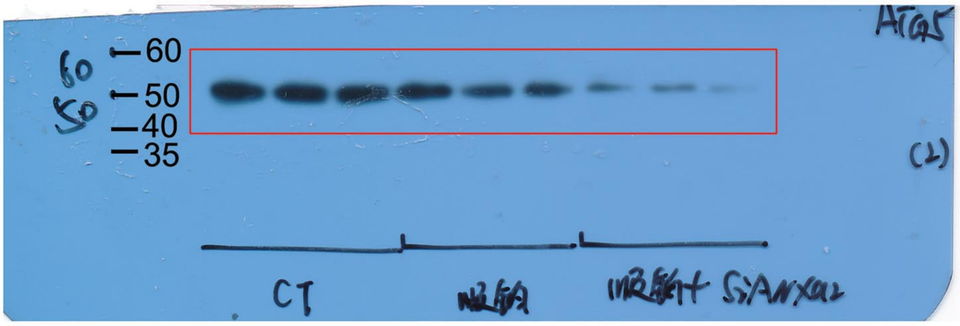

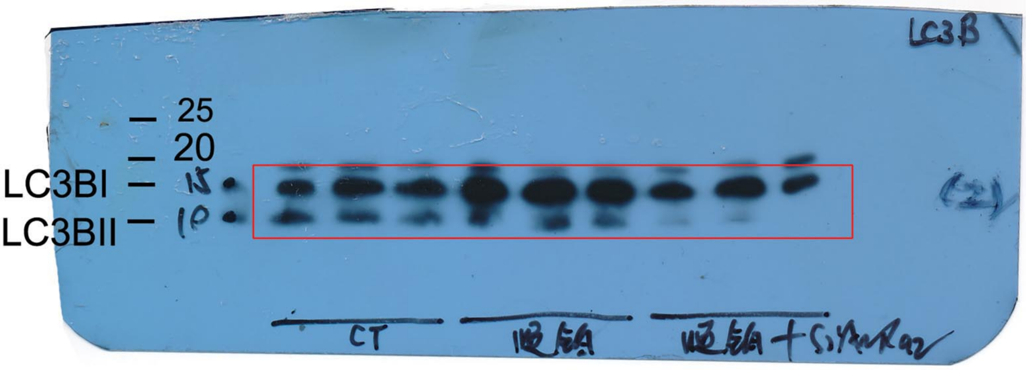

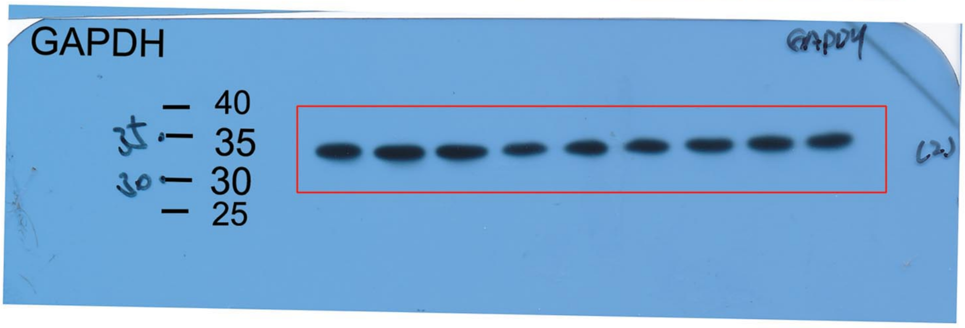

Full unedited gels for Figure 7D

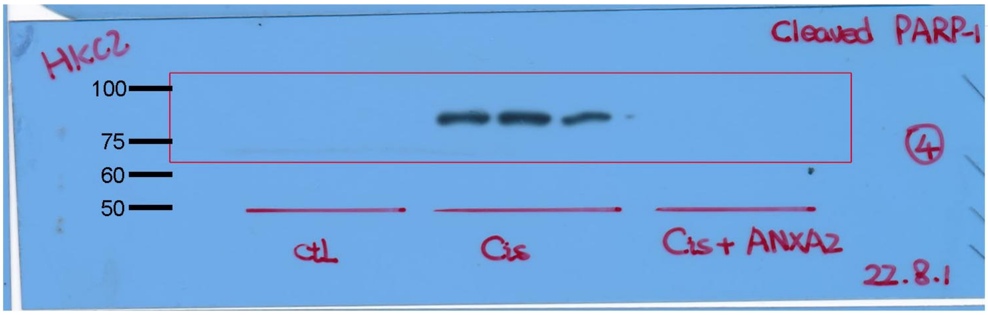

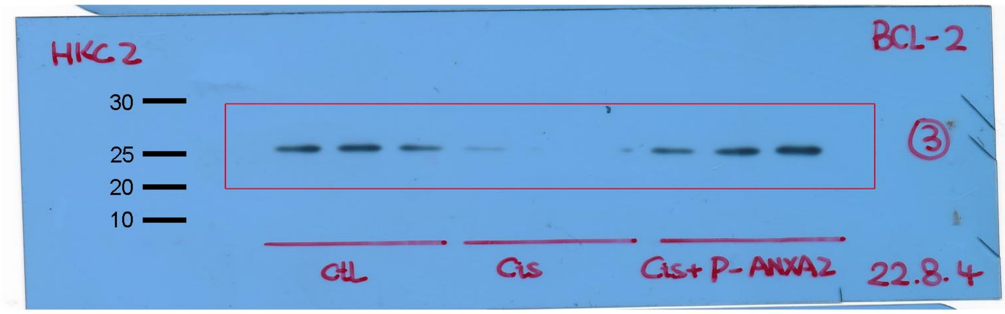

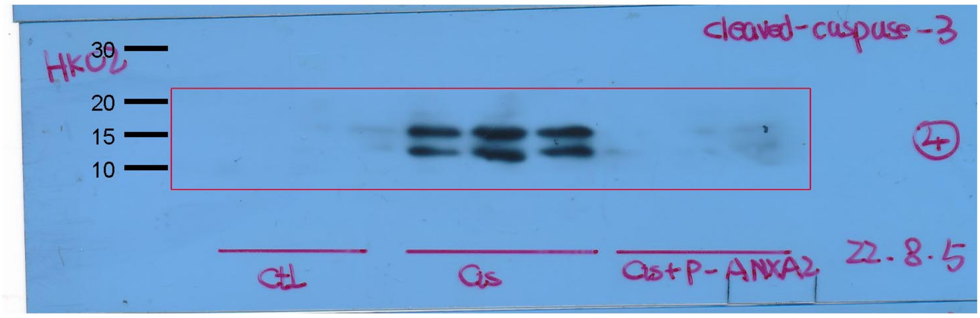

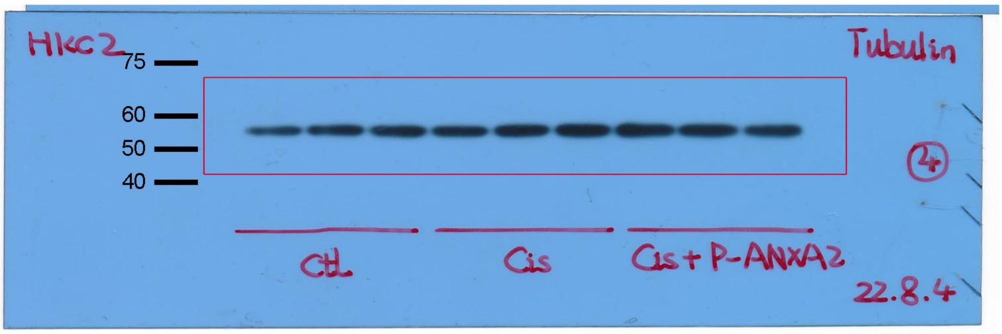

Full unedited gels for Figure 7H

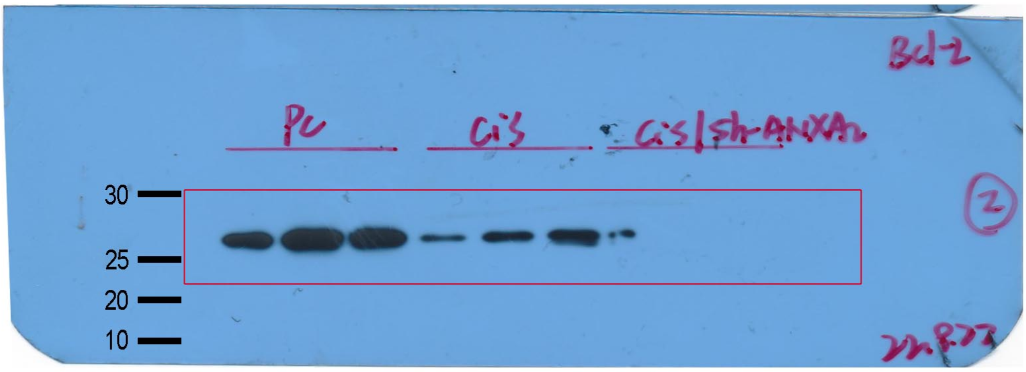

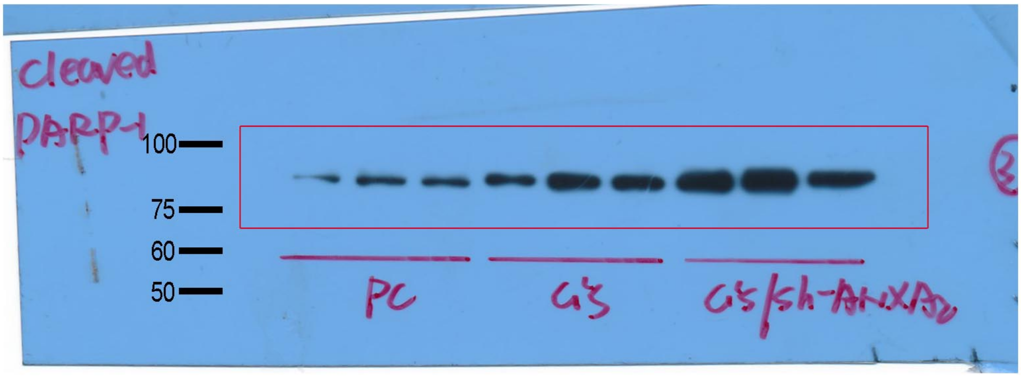

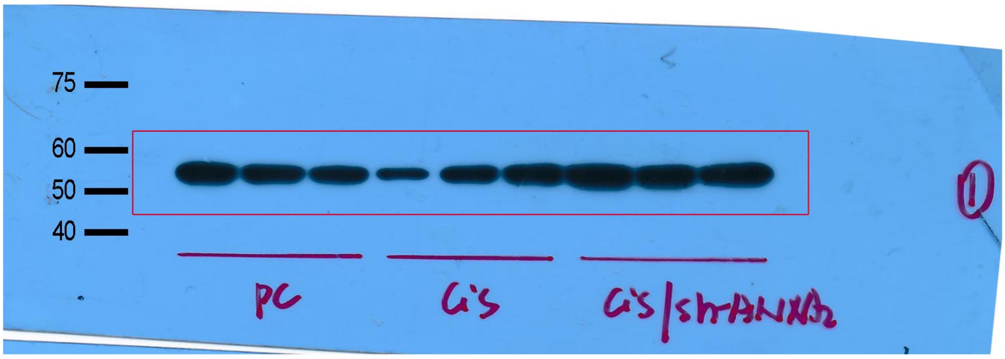

Full unedited gels for Figure 7N

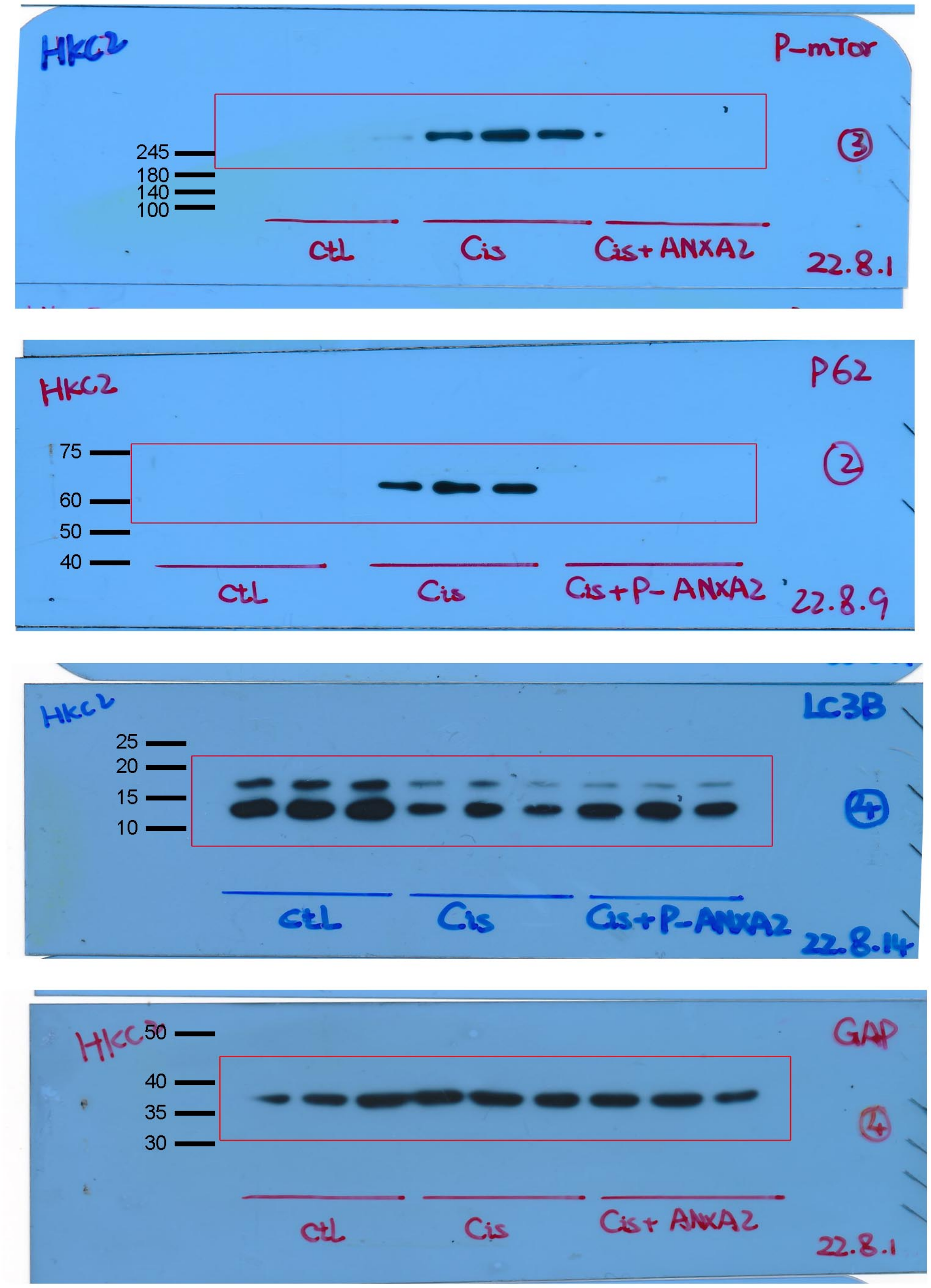

Full unedited gels for Figure 8G

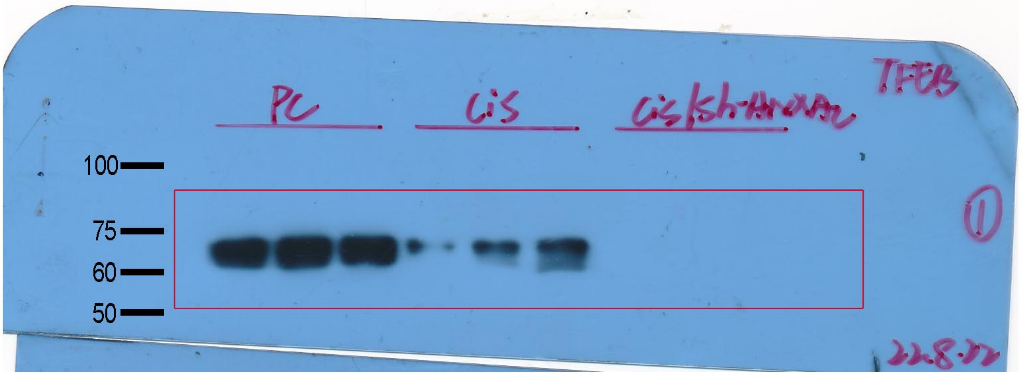

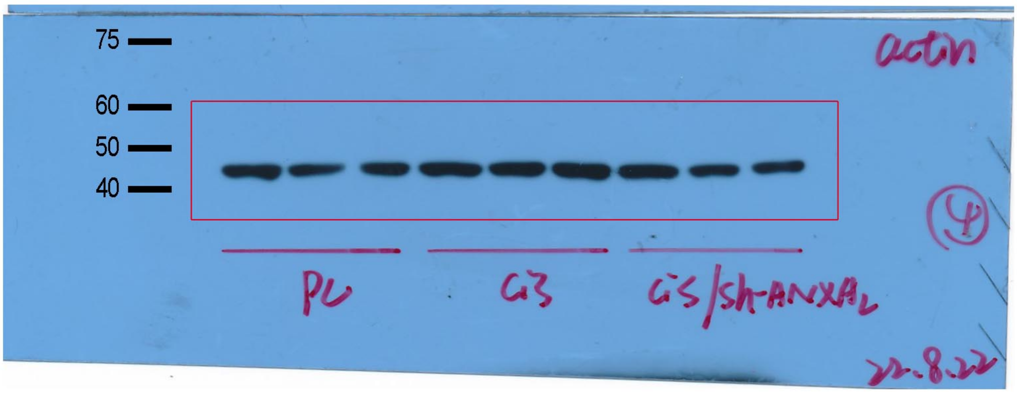

Full unedited gels for Figure 9A

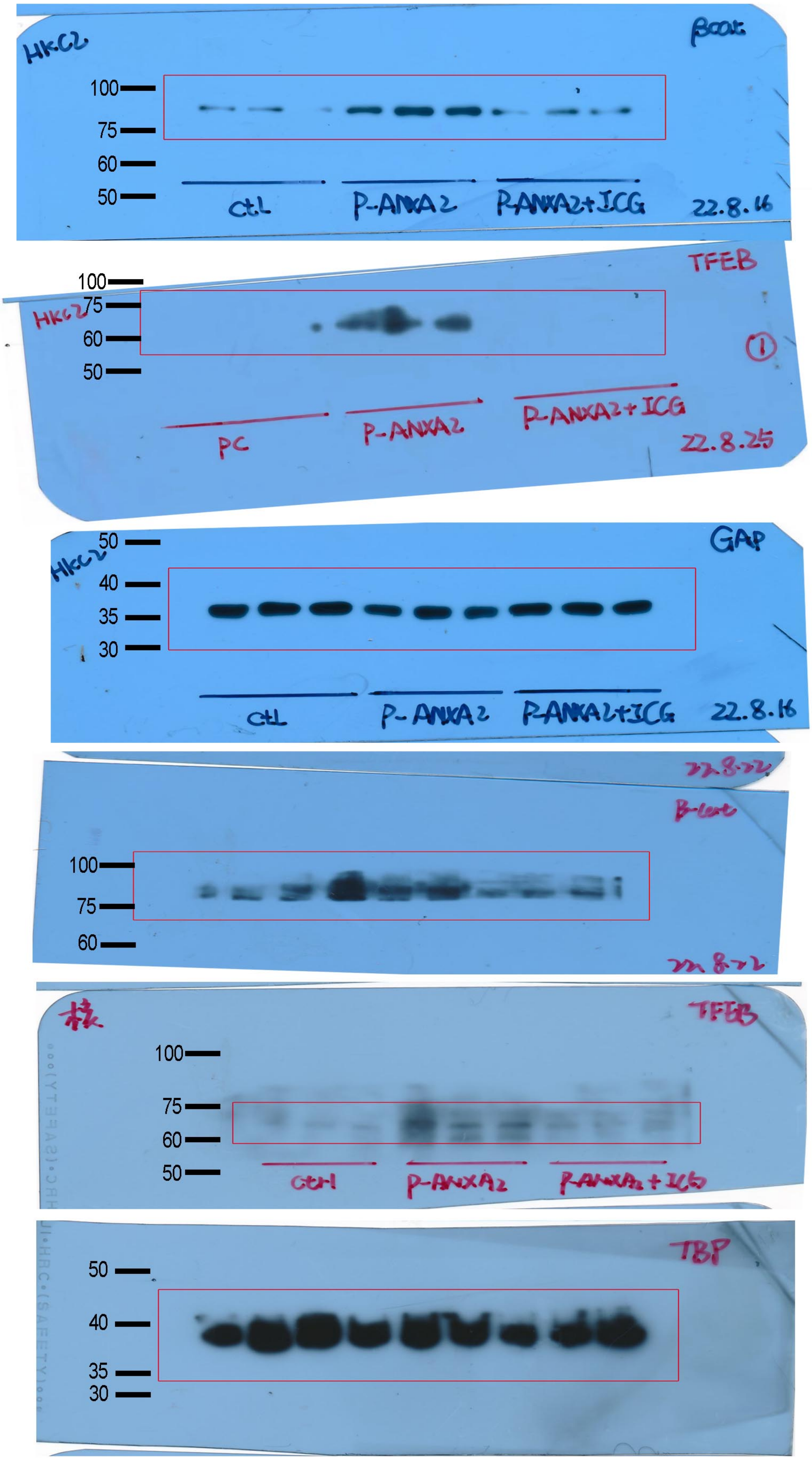

Full unedited gels for Figure 9I

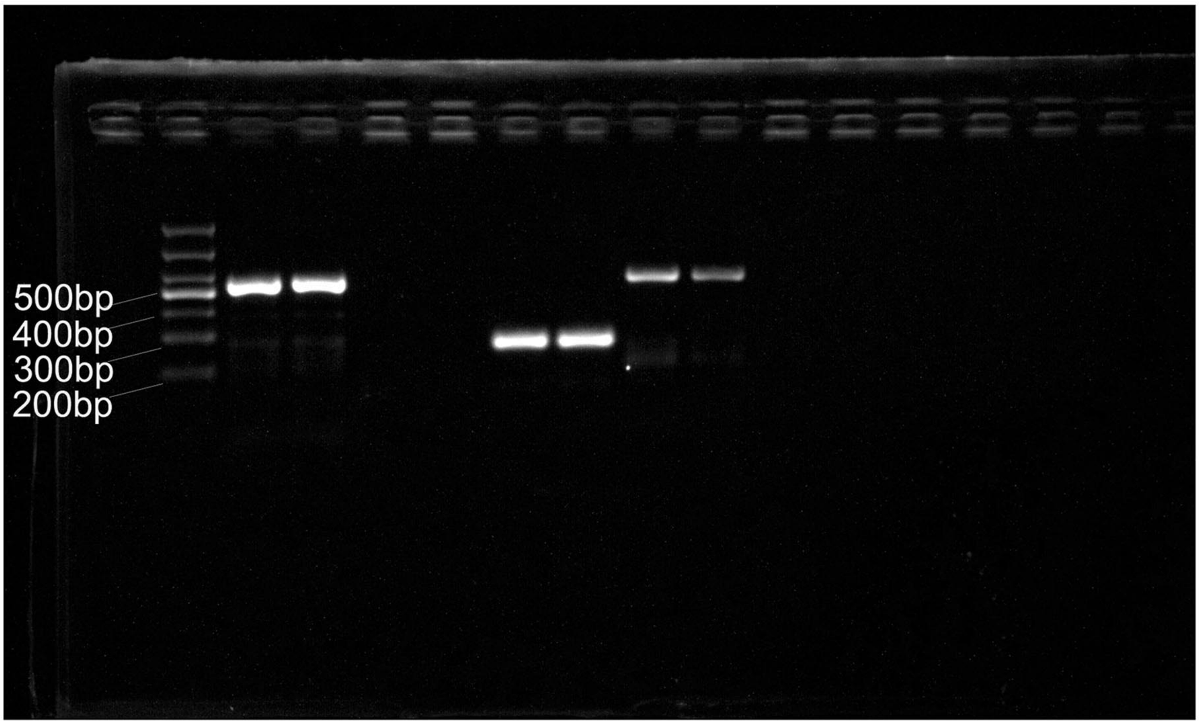

Supplement: Supplementary file 3 — Original Data File [file 41420_2022_1224_MOESM3_ESM.pdf]
